# Mechanism of protein-primed template-independent DNA synthesis by Abi polymerases

**DOI:** 10.1093/nar/gkac772

**Published:** 2022-09-15

**Authors:** Małgorzata Figiel, Marta Gapińska, Mariusz Czarnocki-Cieciura, Weronika Zajko, Małgorzata Sroka, Krzysztof Skowronek, Marcin Nowotny

**Affiliations:** Laboratory of Protein Structure, International Institute of Molecular and Cell Biology, Warsaw, Poland; Laboratory of Protein Structure, International Institute of Molecular and Cell Biology, Warsaw, Poland; Laboratory of Protein Structure, International Institute of Molecular and Cell Biology, Warsaw, Poland; Laboratory of Protein Structure, International Institute of Molecular and Cell Biology, Warsaw, Poland; Laboratory of Protein Structure, International Institute of Molecular and Cell Biology, Warsaw, Poland; Biophysics and Bioanalytics Facility, International Institute of Molecular and Cell Biology, Warsaw, Poland; Laboratory of Protein Structure, International Institute of Molecular and Cell Biology, Warsaw, Poland

## Abstract

Abortive infection (Abi) is a bacterial antiphage defense strategy involving suicide of the infected cell. Some Abi pathways involve polymerases that are related to reverse transcriptases. They are unique in the way they combine the ability to synthesize DNA in a template-independent manner with protein priming. Here, we report crystal and cryo-electron microscopy structures of two Abi polymerases: AbiK and Abi-P2. Both proteins adopt a bilobal structure with an RT-like domain that comprises palm and fingers subdomains and a unique helical domain. AbiK and Abi-P2 adopt a hexameric and trimeric configuration, respectively, which is unprecedented for reverse transcriptases. Biochemical experiments showed that the formation of these oligomers is required for the DNA polymerization activity. The structure of the AbiK–DNA covalent adduct visualized interactions between the 3′ end of DNA and the active site and covalent attachment of the 5′ end of DNA to a tyrosine residue used for protein priming. Our data reveal a structural basis of the mechanism of highly unusual template-independent protein-priming polymerases.

## INTRODUCTION

The coexistence of bacteria and phages leads to a perpetual battle between them. Consequently, bacteria evolved a wide range of anti-phage defense strategies that act at different stages of phage infection (1). Various mechanisms prevent phage entry by blocking phage adsorption to the cell surface or by inhibiting the injection of viral DNA into the cell. Other well-studied mechanisms target and degrade genomic DNA of the invading phage. These include restriction–modification and CRIPSR-Cas systems (2,3). The latter can also provide a means for adaptive immunity of the bacterial cell against pathogens that were previously encountered by its ancestors. Alternatively, the bacterial cell can respond to infection using toxin–antitoxin or abortive infection systems that trigger dormancy, temporary growth arrest or even cell death (4,5).

Abortive infection (Abi) is an altruistic process of programmed cell death that prevents the release of functional virions and thus their spread to other bacterial cells in the population. Most Abi systems that have been identified to date have been characterized in *Escherichia coli* and *Lactococcus lactis* (1). Protein effectors that are involved in these systems are usually plasmid-encoded. They have very diverse activities and thus are assumed to trigger cell death through different mechanisms. Among these are three systems that rely on proteins that are related to reverse transcriptases (RTs): AbiA, AbiK and Abi-P2.

The AbiK system in *L. lactis* is encoded by a single, constitutively transcribed gene that is located on the native pSRQ800 plasmid (6). It provides the host with resistance against 936 and P335 phage species and to some extent c2 species. It has been reported to be capable of non-templated DNA synthesis, an activity that resembles a terminal deoxynucleotidyl transferase (7). The DNA product is covalently bound to the enzyme, indicating protein-primed DNA synthesis (7). The exact mechanism by which AbiK performs its abortive infection action is unknown. However, some phages were found to gain resistance to AbiK through suppressive mutations of sensitivity to AbiK (*sak*) genes. Some *sak* gene products are single-strand annealing proteins that are related to RAD52 and Erf (8).

Abi-P2 proteins are encoded by P2-like prophages (e.g. P2-EC30 and P2-EC58) in *Escherichia coli* (9). Open reading frames that encode the enzyme (*orf570* of P2-EC30 and *orf544* of P2-EC58) are located in a variable region that is believed to be acquired by horizontal transfer (9). The *orf570* gene product was shown to provide the host cell with resistance to phage T5 infection.

Based on sequence homology, Abi DNA polymerases are classified as RTs. Prokaryotic RTs are a vast and diverse group of enzymes (10). Majority of these RTs fall into one of the three main classes defined based on the type of genetic elements that encode them: retron/retron-like sequences, group II introns and diversity-generating retroelements (DGRs). Other minor lineages include RTs associated with CRISPR-Cas, RTs from the so-called ‘unknown-groups’, and group-II-like uncharacterized RTs. Enzymes from many of these groups are involved in anti-phage defense mechanisms.

Protein priming is a mechanism of DNA synthesis initiation in which the first nucleotide of the nascent DNA chain is attached to a hydroxyl group provided by a specific serine, threonine or tyrosine residue of the polymerase or a dedicated protein. The phenomenon of protein-primed DNA synthesis has been observed for several viral DNA polymerases, including enzymes of *Bacillus* bacteriophage phi29 and adenoviruses, both belonging to the B-family of DNA polymerases (11,12). In these systems, the serine residue that is utilized for protein priming is provided *in trans* by a terminal protein (TP). Protein priming is also employed by hepadnaviruses. In this case, the RNA-dependent DNA polymerase and terminal protein domains are both located on one polypeptide chain, called the P protein, and thus protein priming occurs *in cis* (13).

The structure and mechanism of action of AbiK and Abi-P2 proteins, particularly the way they execute protein-primed and template-independent DNA synthesis, are unknown. The present study determined the structures of AbiK and Abi-P2 proteins by a combination of cryo-electron microscopy (EM) and X-ray crystallography. We found that AbiK and Abi-P2 are both composed of two domains (RT-like and helical domains) and adopt a trimeric arrangement that is unprecedented for RT proteins. Based on a series of structures, we elucidated the mechanism of protein priming and template-independent DNA synthesis.

## MATERIALS AND METHODS

### Expression and purification of AbiK

The synthetic gene that coded for *L. lactis* AbiK (*Ll-*AbiK) was purchased from BioBasic and subcloned into a pET28 expression vector that carried an N-terminal His6-SUMO-tag that is removable by SUMO protease. Recombinant proteins were expressed in *E. coli* BL21 Star (DE3) cells in Luria-Bertani Broth or Super Broth (Formedium) supplemented with glycerol. Protein expression was induced overnight with 0.4 mM isopropyl 1-thio-β-D-galactopyranoside at 18°C. Bacterial cells were next suspended in 40 mM NaH_2_PO_4_ (pH 7.0), 100 mM NaCl, 5% glycerol and 10 mM imidazole with the addition of a mixture of protease inhibitors and viscolase and incubated on ice in the presence of 1 mg/ml lysozyme. After sonication, the cleared lysate was applied to a HisTrap column (GE Healthcare) that was equilibrated with 10 mM imidazole, 40 mM NaH_2_PO_4_ (pH 7.0), 0.5 M NaCl and 5% glycerol. After wash steps with 60 and 180 mM imidazole, the protein was eluted with 300 mM imidazole. Selected fractions were dialyzed overnight against 10 mM imidazole, 40 mM NaH_2_PO_4_ (pH 7.0), 0.5 M NaCl, and 5% glycerol in the presence of SUMO protease. The sample was then reapplied to the HisTrap column under the same conditions. Cleaved protein did not bind to the resin and was collected in the flow-through fraction, which was next applied to a Superdex 200 Increase 10/300 GL gel filtration column that was equilibrated with 20 mM HEPES (pH 7.0), 400 mM NaCl, 0.5 mM ethylenediaminetetraacetic acid (EDTA) and 1 mM dithiothreitol (DTT). Fractions that contained pure protein were pooled and concentrated.

### Expression and purification of Abi-P2

The synthetic gene that coded for Abi-P2 (residues 1–541) of *E. coli* prophage EC30 was purchased from BioBasic and subcloned into a pET28a-SUMO expression vector that contained an N-terminal His6-SUMO-tag that is removable by SUMO protease. Initially, a full-length protein (1–546) was purified and crystallized. The crystals, however, did not diffract X-rays to a sufficient resolution. A slightly shortened 1–541 variant produced better quality crystals and allowed us to collect a dataset suitable for structure solution. Recombinant proteins were expressed in *E. coli* BL21 Gold (DE3) cells. The pellet from bacteria that expressed the desired protein was suspended in 50 mM Tris (pH 7.0), 150 mM NaCl, 20 mM imidazole, 5% glycerol and 5 mM 2-mercaptoethanol with the addition of a mixture of protease inhibitors, lysozyme, and viscolase and incubated on ice. After sonication, the cleared lysate was loaded onto a HisTrap HP column (GE Healthcare) that was equilibrated with 20 mM imidazole, 25 mM Tris (pH 7.0), 0.5 M NaCl, 5% glycerol, and 5 mM 2-mercaptoethanol. After a 150 mM imidazole wash, the protein was eluted with 400 mM imidazole. Selected fractions were dialyzed overnight against 20 mM imidazole, 25 mM Tris (pH 7.0), 0.5 M NaCl, 5% glycerol and 5 mM 2-mercaptoethanol in the presence of SUMO protease. The sample was then reapplied to the HisTrap column under the same conditions. Cleaved protein did not bind to the resin and was collected in the flow-through fraction, which was next concentrated and applied to a Superdex 200 Increase 10/300 GL gel filtration column that was equilibrated with 25 mM Tris (pH 7.0), 400 mM NaCl, 5% glycerol and 1 mM DTT. Fractions that contained pure protein were pooled and concentrated.

### Cryo-EM sample preparation and data collection

*Ll-*AbiK (3 μl, 1.0 mg/ml) was applied to a glow-discharged Quantifoil R1.2/1.3 mesh 200 Cu grid and vitrified in liquid ethane with an FEI Vitrobot Mark IV (Thermo Fisher Scientific) at 4°C with 95% humidity and 4 s blot time. The grid was imaged with a Glacios electron microscope (Thermo Fisher Scientific) that operated at 200 kV and was equipped with a Falcon 3EC camera at the Centre of New Technologies, University of Warsaw. A total of 1429 movies were recorded in counting mode with a physical pixel size of 0.92 Å (nominal magnification of 150 000× ; refined further to 0.95 Å as described below), 100 μm objective aperture, and nominal defocus range of −1.6 μm to −0.6 μm (with 0.2 μm steps). The total dose (fractionated into 40 frames) was 40 e/Å^2^, and the dose rate was 0.87 e/pixel/s.

*Ll-*AbiK Y44F (3 μl, 1.74 mg/ml) was applied to a glow-discharged C-flat-2/1 mesh 200 Cu grid and vitrified in liquid ethane with an FEI Vitrobot Mark IV (Thermo Fisher Scientific) at 4°C with 95% humidity and 4 s blot time. Data collection was performed on a Titan Krios G3i electron microscope (Thermo Fisher Scientific) that operated at 300 kV and was equipped with a BioQuantum energy filter (with 20 eV energy slit) and K3 camera (Gatan) at the SOLARIS National Synchrotron Radiation Centre (Krakow, Poland). A total of 5063 movies were recorded with 20° and 30° stage tilt in counting mode with a physical pixel size of 0.86 Å (nominal magnification of 150 000× ; refined further to 0.82 Å as described below), 50 μm C2 condenser aperture, and retracted objective aperture. Nominal defocus range was −2.5 μm to −1.5 μm. The total dose (fractionated into 40 frames) was 60 e/Å^2^, and the dose rate was 16.55 e/pixel/s.

Abi-P2 was diluted in 20 mM Tris (pH 7.0), 500 mM NaCl, and 1 mM DTT to a concentration of 1 mg/ml and vitrified on a Quantifoil R2/1 mesh 200 Cu grid under the same conditions as *Ll*-AbiK. Data collection was performed on a Titan Krios G3i electron microscope at the SOLARIS National Synchrotron Radiation Centre (Krakow, Poland). A total of 1348 movies were recorded with 40°, 50° and 60° stage tilt and a nominal defocus of −2.0, −1.5 and −1.0 μm. Movies were collected with aberration-free image shift (AFIS) at a nominal magnification of 105 000× (corresponding to a physical pixel size of 0.86 Å), 50 μm C2 condenser aperture, and retracted objective aperture. The total dose (fractionated into 40 frames) was 61 e/Å^2^, and the dose rate was 16.26 e/pixel/s.

### Cryo-EM data processing and interpretation

Cryo-EM images were processed with RELION-3.1 (14) and cryoSPARC 3.2 (15). For the *Ll-*AbiK dataset (Supplementary Figure S1), raw movies were converted to tiff format with the *relion_convert_to_tiff* script, motion-corrected, and binned 2× using RELION’s implementation of MotionCor2 software (16). The contrast transfer function (CTF) was fitted with CTFFIND4.1 (17). A total of 748 962 particles were selected with the Laplacian-of-Gaussian algorithm implemented in RELION, binned 2× to a pixel size of 3.68 Å/pix, and subjected to two rounds of reference-free 2D classification. A total of 308 909 selected particles were re-extracted with a pixel size of 1.84 Å/pix and used to generate *de novo* a 3D initial model with the Stochastic Gradient Descent (SGD) method implemented in RELION. The generated model was aligned to D3 symmetry axes and 3D refined with imposed D3 symmetry which resulted in a 3.68 Å reconstruction. Bayesian polishing (with re-extraction with an unbinned pixel size of 0.92 Å/pix), followed by CTF refinements, improved resolution to 2.6 Å. After the second round of Bayesian polishing, ‘shiny particles’ (i.e. particles with improved signal-to-noise ratio after local motion estimation) were imported to cryoSPARC and subjected to 2D classification, after which 227 461 particles were selected. Non-uniform refinement with imposed D3 symmetry, as well as local and global CTF refinements, produced a map with a resolution of 2.23 Å. Comparison of this map with the crystallographic model allowed for the precise pixel size calibration. To this end, a series of EM maps with altered pixel size (modified with the *alterheader* script from IMOD package (18)) were compared with the crystallographic model by using the *Fit in Map* tool in UCSF Chimera (19). Resulting correlation values were plotted against the pixel size (Supplementary Figure S2). Selected particles were re-imported to cryoSPARC with refined pixel size of 0.95 Å and subjected to CTF refinements. Final non-uniform refinement resulted in the reconstruction with the resolution of 2.3 Å (Supplementary Figure S3). The final map was sharpened locally with the Local Filter tool in cryoSPARC. This cryo-EM map of *Ll-*AbiK was used for *de novo* model building with Buccaneer (20). The initial model was manually edited in Coot (21) and refined using real space refinement in Phenix (22). The whole length of *Ll-*AbiK polypeptide chain is visible in the model except for a loop comprising residues 189–190. Eleven nucleotides of the DNA chain could be traced in each of the subunits. Because untemplated DNA synthesis by AbiK results in a random sequence, arbitrarily chosen poly-dC DNA was built in the complex model.

For AbiK Y44F (Supplementary Figure S4), raw movies were motion-corrected and binned 2x using RELION’s implementation of MotionCor2 software, and the CTF was fitted with CTFFIND4.1 (17). A total of 2 306 347 particles were picked with crYOLO software (23) and subjected to two rounds of reference-free 2D classification in cryoSPARC. In line with the observation that in solution *Ll*-AbiK Y44F variant is predominantly monomeric, a majority of the particles corresponded to monomers, however a fraction of trimeric and hexameric particles was also observed. Initial attempts to solve the *Ll*-AbiK Y44F structure based on monomeric particles yielded a low quality reconstruction that suffered from strong orientational bias. Since hexameric particles were present in a wider range of orientations, for the final reconstruction we selected only the 2D classes that represented hexamers. The wild-type *Ll*-AbiK reconstruction low-pass filtered to 30 Å was used as an initial model for the 3D refinement. A total of 59 721 particles were selected and subjected to 3D refinement with imposed D3 symmetry, resulting in a 4.17 Å reconstruction. Importantly, we did not observe any significant differences between the density for a single subunit of *Ll*-AbiK Y44F hexamer and the preliminary reconstruction of the monomer. To improve the quality of our hexameric reconstruction, the particles were re-imported into RELION with scripts from UCSF pyem and subjected to Bayesian polishing (with re-extraction with an unbinned pixel size of 0.86 Å/pix). ‘Shiny particles’ were again imported to cryoSPARC and subjected to a final round of 2D classification. A non-uniform 3D refinement using the selected 50 340 particles improved the resolution to 3.05 Å. After the second round of Bayesian polishing in RELION the pixel size was refined to 0.82 Å using an approach described above for wild-type AbiK reconstruction (Supplementary Figure S5). Final non-uniform refinement in cryoSPARC resulted in a reconstruction with the resolution of 2.7 Å. The final map was sharpened locally with the Local Filter tool in cryoSPARC. Structure of the wild-type AbiK was used as an initial model for real space refinement in Phenix and the resulting model was manually edited in Coot. Almost whole length of the polypeptide chain is visible in the model except for regions 32–42 and 189–191.

For Abi-P2 (Supplementary Figure S6), raw movies were motion-corrected and binned 2x using RELION’s implementation of MotionCor2 software, and the CTF was fitted globally with CTFFIND4.1 (17). A total of 614 818 particles were picked with the Laplacian-of-Gaussian algorithm that was implemented in RELION software, binned 2× to a pixel size of 3.44 Å/pixel, and subjected to reference-free 2D classification in cryoSPARC. A total of 93 444 selected particles were used to generate *de novo* a 3D initial model with the SGD method implemented in cryoSPARC. The generated model was 3D refined with imposed C3 symmetry which resulted in a 7.3 Å reconstruction. Refined particles were re-imported into RELION with scripts from UCSF pyem, re-extracted with a pixel size of 1.72 Å/pixel, and subjected to 3D refinement with C3 symmetry, resulting in a 4.6 Å reconstruction with strong orientational bias. Subsequent Bayesian polishing (with re-extraction with an unbinned pixel size of 0.86 Å/pixel), followed by CTF refinements, improved resolution to 3.9 Å. The quality of the reconstruction was still too low to permit *ab initio* tracing of the polypeptide chain and thus it was only used for phasing the X-ray diffraction data and has not been deposited in EMDB. The final map was sharpened in RELION with a *B* factor of −30 Å^2^. For all structures resolution was estimated from gold-standard-masked FSC at the 0.143 threshold.

### Protein crystallization

The final concentration of *Ll*-AbiK that was used for crystallization was 4.5 mg/ml. The protein was mixed with the reservoir solution and diluted crystal seeds that were grown in 30% PEG 400, 0.2 M MgCl_2_, 0.1 M HEPES (pH 7.5) and crystallized using the sitting drop vapor diffusion method at 18°C. Crystals were obtained with 30% Jeffamine M-600, 0.1 M MES (pH 6.5), and 0.05 M cesium chloride. For data collection, the crystals were flash frozen in liquid nitrogen.

The final concentration of Abi-P2 that was used for crystallization was 6 mg/ml. Crystals that produced the best X-ray diffraction were obtained under two conditions: [6% (v/v) 2-propanol, 26% PEG MME 550, 0.1 M sodium acetate trihydrate (pH 4.5)] and [0.1 M sodium malonate (pH 8.0), 0.1 M Tris (pH 8.0), 30% PEG 1000]. For crystallization, the sitting drop vapor diffusion method was used at 18°C and 4°C. For data collection, the crystals were flash frozen in liquid nitrogen.

### X-ray diffraction data collection and structure determination

The X-ray diffraction data for *Ll-*AbiK crystals were collected at the PETRA III storage ring at beamline P14. The dataset was processed and scaled using XDS/XDSAPP GUI (24). The *Ll*-AbiK crystals belonged to the *P*6_2_ 2 2 spacegroup and contained six molecules of AbiK in the asymmetric unit arranged in the form of a hexamer. The structure was solved with Phenix Phaser-MR (25) using the preliminary structure that was traced into cryo-EM maps as the search model. The nucleic acid model was manually built into the structure in Coot (21). Because untemplated DNA synthesis by AbiK results in a random sequence, arbitrarily chosen poly-dC DNA was built in the complex model. Refinement of the structure was performed with Phenix (22), interspersed with rounds of manual correction in Coot, and 1.1% of the reflections were used to calculate *R*_free_. In the final model, 99.8% of the residues reside in the allowed regions of the Ramachandran plot. The only Ramachandran outlier in all subunits of both structures is Val246, however its unusual conformation is strongly supported by the cryo-EM density map. The whole length of *Ll-*AbiK polypeptide chain is visible in the structure except for one loop comprising residues 189–190, which is missing in most subunits. Eight to ten nucleotides of the DNA chain could be traced in individual subunits.

The X-ray diffraction data for Abi-P2 were collected at the PETRA III storage ring at the P11 DESY beamline. The crystals belonged to the *P*2_1_ spacegroup and contained six molecules of Abi-P2 in the asymmetric unit arranged in the form of two trimers. Data were processed and scaled using XDS. The structure was solved by molecular replacement with a low-resolution cryo-EM density map for Abi-P2 as the search model using the Phenix Phaser-MR (25) as described previously (26). Initial model was built with Buccaneer (20) and an AlphaFold2 (27) model was used to improve manual Abi-P2 chain building in the C-terminal region. The asymmetric unit contained six protein molecules in a dimer-of-trimers configuration. The structure was refined in phenix.refine (22) with manual building in Coot (21). In the final model, all residues except for one reside in the allowed regions of the Ramachandran plot. Out of 541 residues of the Abi-P2 variant that was used, residues 32–541 are visible in the structure with the exception of loops 159–169, 214–222, 238–240 and 465–468.

### Activity tests

Template-independent DNA polymerization reactions were performed for *Ll-*AbiK (wild-type and substitution variants) at a concentration of 0.5 μM in the presence of a deoxynucleotide mix (100 μM of each dNTP), 5 μM fluorescein-dUTP (Thermo Scientifc, R0101), and 2 mM MgCl_2_, in buffer that contained 50 mM Tris (pH 8.5), 100 mM NaCl, and 5 mM DTT. The reactions were incubated at 37°C for 5, 10 or 15 min and stopped by the addition of 40 mM EDTA. The samples were next treated with proteinase K to remove the enzyme. All reactions were performed in triplicate. Polymerization products were analyzed on 10% denaturing Tris borate EDTA (TBE)-urea polyacrylamide gels and visualized by fluorescence readout using Amersham Typhoon (Cytiva) biomolecular imager. To generate DNA size marker ΦX174 DNA (New England BioLabs, N3023S) was amplified by PCR in the presence of fluorescein-dUTP and the PCR product was digested with HaeIII restriction enzyme producing fragments of 72–1353 nucleotides. Fluorescein-labeled 18 nt synthetic DNA was used as an additional size marker. For densitometry analysis, bands that corresponded to long DNA products on scanned gels were quantified with ImageQuant software (Cytiva). We note that given the very high difference in intensity between the band of free fluorescein-dUTP and that of the DNA product it was not possible to reliably correct for loading errors by quantitating the signal coming from the product relative to total fluorescent signal in each lane. Instead, signal from long product was normalized against the signal from two bands that corresponded to the contaminants of fluorescein-dUTP, whose intensity was proportional to the amount of the labeled nucleotide used in the reaction. The resulting values were normalized relative to the amount of product observed for wild-type *Ll*-AbiK after 15 min of reaction which was expressed as 100%. Normalized values were plotted with mean ± SD using GraphPad Prism software (Dotmatics). Statistical significance of the difference in activity between the wild-type and each enzyme variant was calculated using paired sample *t*-test.

Polymerase assays for Abi-P2 were performed with protein at 1 μM concentration and 2 μM Texas Red-dCTP (Jena Bioscience, NU-809-TXR-S), in a reaction that contained 50 mM Tris (pH 8.5), 150 mM KCl, 2 mM MgCl_2_, 5 mM DTT and 100 μM deoxynucleotide mix. The reactions were incubated at 37°C for 5, 15, 60 and 120 min and stopped by adding a solution with EDTA and proteinase K. All reactions were performed in triplicate. Samples were analyzed on 10% TBE-urea polyacrylamide gels and fluorescent products of the reactions were visualized using Amersham Typhoon biomolecular imager. Three molecular size markers – respectively 20, 50 and 100 nt in size—were used to assess polymerization products’ size. 20 nt and 50 nt markers were synthetic oligonucleotides labeled with Texas Red ordered from Eurofins Genomics. 100 nt marker was produced by PCR amplification of 100-nt long DNA sequence in the presence of Texas Red-dCTP. For densitometry analysis, bands that corresponded to DNA products on scanned gels were quantified with ImageQuant software (Cytiva). To correct for loading errors, activity of Abi-P2 variants was expressed as percentage of signal from the products relative to total fluorescent signal in each lane. The resulting values were normalized relative to the amount of product observed for wild-type Abi-P2 after 120 min of reaction which was expressed as 100%. These values were then plotted with mean ± SD using GraphPad Prism software (Dotmatics). Paired sample *t*-test was used to assess the significance of the differences in activity between variants.

### Multi-angle light scattering (MALS)

Molecular masses of native protein complexes were measured by SEC-MALS using a system consisting of Akta Purifier liquid chromatography system (Cytiva) with inline Dawn 8+ MALS detector (Wyatt) and Optilab T-rEX refractive index detector (Wyatt) on Superdex 200 Increase 10/300 GL Column (Cytiva). 100 μl samples were applied to the column equilibrated with 20 mM HEPES (pH 7.0), 400 mM NaCl, 0.5 mM EDTA and 1 mM DTT (*Ll*-AbiK) or in 25 mM Tris (pH 7.0), 400 mM NaCl, 2% glycerol and 1 mM DTT (Abi-P2). Molecular masses were assessed in Astra 6 software (Wyatt) using Zimm model. dRI was used for inline protein concentration measurement and dn/dc = 0.185 was used for calculations.

### Liquid chromatography–mass spectrometry (LC–MS)

Direct infusion experiments were performed on a Waters (Milford, MA, USA) Synapt G2 quadrupole time-of-flight hybrid mass spectrometer in the positive ion electrospray ionization mode via the instrument's built-in fluidics system. LC–MS experiments were performed using Waters Acquity UPLC system coupled to the same mass spectrometer. The TOF-MS tuning parameters were as follows: capillary voltage 2.5 kV, source temperature 80°C, desolvation temperature 150°C, and desolvation gas flow 650 l/h. UPLC separation was performed using a Waters Acquity UPLC Protein BEH C4 column (300 Å, 1.7 μm, 1 mm × 50 mm), with 0.1% formic acid in water as mobile phase A and 0.1% formic acid in acetonitrile as mobile phase B. Results were analyzed using MassLynx software.

### Fourier-transform infrared spectroscopy (FT-IR)

Secondary structure analysis for wild-type *Ll*-AbiK and its T151W/T369W variant was performed using a Bruker Tensor 27 FT-IR spectrometer. Protein samples were used at a concentration of 1.7 mg/ml in a buffer that contained 20 mM HEPES (pH 7.0), 400 mM NaCl, 0.5 mM EDTA and 1 mM DTT. The spectra were analyzed using OPUS-QUANT2 software with the protein spectrum library that was provided by the supplier as a reference.

#### Reagents

Cryo-EM samples were prepared on Quantifoil R1.2/1.3 mesh 200 Cu grids, Quantifoil R2/1 mesh 200 Cu grids (Quantifoil Micro Tools GmbH, Großlöbichau, Germany), and C-flat-2/1 mesh 200 Cu grids (Protochips, Morrisville, NC, USA) using Q150T ES glow discharge system (Quorum Technologies, East Sussex, England) and Vitrobot Mark IV (Thermo Fisher Scientific, MA, USA). Protein crystals were produced using the following crystallization kits: Crystal Screen (Hampton Research, HR2-110), PEGRx 2 (Hampton Research, HR2-084) and crystallization robots: Phoenix (Art Robbins Instruments, Sunnyvale, CA, USA), Oryx (Douglas Instruments, Hungerford, UK). For biochemical experiments the following fluorescently labeled deoxynucleotides were used: Fluorescein-dUTP (Thermo Scientifc, R0101), Texas Red-dCTP (Jena Bioscience, NU-809-TXR-S). The results of activity assays were visualized by fluorescence readout using Amersham Typhoon (Cytiva, Marlborough, MA, USA) biomolecular imager.

#### Biological resources

The following *Escherichia coli* strains were used in this study: BL21 Star (DE3) [F- *ompT hsdSB* (r_B_^−^ m_B_^–^) *gal dcm*^+^*rne131* (DE3)], BL21 Gold(DE3) [B F^−^*ompT hsdS*(r_B_^−^ m_B_^–^) *dcm*^+^ Tet^r^*gal* λ(DE3) *endA* Hte], Top10 [F^−^*mcrA* Δ(*mrr-hsdRMS-mcrBC*) φ80lacZΔM15 ΔlacX74 recA1 araD139 Δ(ara-leu)7697 galU galK rpsL (Str^r^) endA1 nupG].

## RESULTS

### *Ll*-AbiK and Abi-P2 structure determination

**Figure 1.**
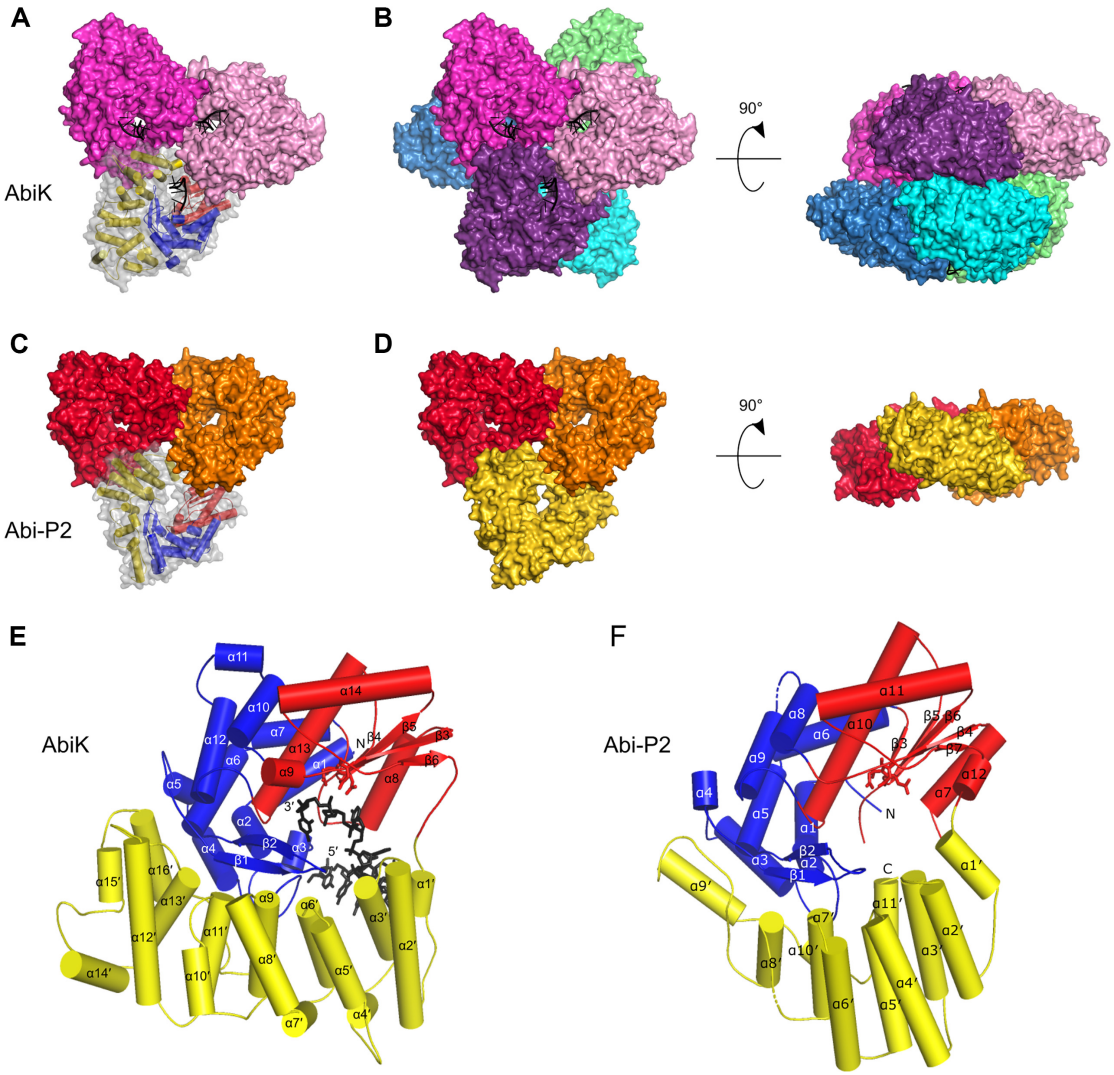
Overall structures of *Ll-*AbiK and Abi-P2 oligomers and monomers. (**A**) Crystal structure of *Ll-*AbiK trimer. The protein is shown in surface representation with subunits shown in different colors. One subunit of the trimer is shown in transparent surface and cartoon representations with the polymerase domain shown in red (palm) and blue (fingers), the helical domain shown in yellow, and DNA shown in black. (**B**) Crystal structure of *Ll-*AbiK hexamer (two views). (**C**) Crystal structure of Abi-P2 shown as in (A). (**D**) Trimer of Abi-P2 (two views). (**E**) Crystal structure of *Ll-*AbiK monomer shown in cartoon representation. The polymerase domain is shown in red for palm and blue for fingers, the helical domain is shown in yellow, and DNA is shown in black. Active site residues are shown as sticks. (**F**) Crystal structure of Abi-P2 monomer shown as in (E).

The structure and mechanism of RT-related abortive infection proteins were previously unknown. Therefore, our goal was to determine the structure of these proteins to explain their mode of action. We chose two distantly related representatives of this group: *L. lactis* AbiK (*Ll*-AbiK) and Abi-P2 enzyme of Enterobacteria phage P2-EC30. Recombinant *Ll*-AbiK was purified after overexpression in *E. coli*. The purified protein exhibited a high ratio of absorbance at 260 and 280 nm wavelengths (Abs_260_/Abs_280_), suggesting the presence of nucleic acid in the sample that could not be removed during purification. Given the reported protein-primed activity of *Ll*-AbiK, we assumed that this nucleic acid is a covalently bound product of DNA synthesis. The structure of *Ll*-AbiK was determined by single-particle cryo-EM at 2.3 Å resolution (Supplementary Figures S1–S3, Supplementary Table S1, Supplementary Table S2). We also obtained crystals of *Ll*-AbiK that diffracted X-rays to 3.1 Å resolution (Supplementary Figure S7, Supplementary Table S3). The polypeptide chain was first traced into the cryo-EM maps, and the resulting model was used to solve the crystal structure by molecular replacement (Figure 1A, B). Refined structures were nearly identical (root-mean-square deviation = 0.351 Å over 545 C-α atoms for a single protomer) (Supplementary Figure S8).

Similarly, Abi-P2 was expressed in bacteria and purified. In contrast to *Ll*-AbiK, the Abs_260_/Abs_280_ ratio of ∼0.6 indicated that no nucleic acid was present in the purified sample. The protein was first subjected to cryo-EM analysis. However, the protein particles on cryo-EM grids exhibited a significant orientational bias, and thus a tilted dataset had to be collected. Data processing resulted in a low-resolution anisotropic cryo-EM reconstruction that did not have quality sufficient for model building (Supplementary Figure S6, Supplementary Table S1). We also obtained Abi-P2 crystals that diffracted X-rays to 3.1 Å. The crystal structure was solved by molecular replacement using the low resolution cryo-EM map (Figure 1C, D, Supplementary Table S3). Most of the subsequent analyses of both *Ll-*AbiK and Abi-P2 structures are based on X-ray crystallography models.

### *Ll*-AbiK and Abi-P2 adopt three-fold symmetry architecture

Both *Ll-*AbiK and Abi-P2 comprise two domains: an N-terminal RT-like domain and a C-terminal helical domain (Figure 1E, F). The RT-like domains of *Ll-*AbiK and Abi-P2 exhibit 31.6% sequence identity and 65.3% sequence similarity and are structurally very similar (root-mean-square deviation = 6.2 Å over 152 C-α atoms). Organization of the RT-like domains corresponds to the canonical ‘right hand’ fold with palm and fingers subdomains. However, the thumb subdomains, which in canonical RTs are involved in proper positioning of the double helical nucleic acid substrate, are absent in both Abi proteins. Instead, the expected position of the thumb subdomain is occupied by N-terminal portions of the helical domains. The palm subdomains of *Ll-*AbiK and Abi-P2 contain a β-sheet of four or five strands, respectively, and four α-helices. The positions of three of the helices (α8, α13 and α14 in *Ll*-AbiK and α7, α10 and α11 in Abi-P2) are very similar between *Ll-*AbiK and Abi-P2. The fingers subdomains of *Ll-*AbiK and Abi-P2 contain ten and eight α-helices, respectively, and two β-strands, the latter forming a β-hairpin. Most regions of fingers subdomains from the two Abi proteins are superimposable.

The C-terminal helical domain is composed of 16 and 11 helices in *Ll-*AbiK and Abi-P2, respectively (Figure 1E, F). The essential role of the additional C-terminal helices of *Ll-*AbiK was demonstrated by the fact that the deletion of 46 C-terminal amino acids eliminated the antiphage activity of *Ll-*AbiK (6). In each of the proteins, the helices of the C-terminal domain are arranged to form an α solenoid that extends from the C-terminus of the palm subdomain toward the fingers subdomain. There is no sequence homology between the two helical domains. Structurally, only the overall shape is retained. A DALI search for similar structures revealed moderate (*Z* factor ≤ 9.0) similarity of the helical domain of *Ll-*AbiK to HEAT repeat domains. In both *Ll-*AbiK and Abi-P2, the RT-like and helical domains form two distinct lobes of the structure that adopt a shape that roughly resembles the letter ‘C’. The space between the lobes serves as a channel where the nascent DNA chain is accommodated.

Early in the cryo-EM image analysis, it became apparent that *Ll-*AbiK formed hexamers, and Abi-P2 formed trimers. The same oligomerization state was also observed in the refined crystal structures of *Ll-*AbiK and Abi-P2. This was in agreement with gel filtration/multiangle light scattering (GF/MALS) experiments, in which *Ll-*AbiK eluted as a peak with a measured molecular weight (MW) of 413.7 kDa (the theoretical MW of a hexamer is 429.3 kDa), and the elution profile of Abi-P2 contained a dominant peak with a MW of 182.6 kDa (the theoretical MW of a trimer is 191.6 kDa; Supplementary Figure S9). The formation of hexamers/trimers is a unique feature of Abi proteins, as no other RTs have been reported to adopt such an architecture. The *Ll-*AbiK hexamer is composed of two trimers that are stacked back-to-back, with the palm subdomains facing inward (Figure 1A, B). In line with D3 symmetry of the hexamer, the two trimers are rotated relative to each other by 60° around a central three-fold axis of the trimer. Within one trimer, the tips of the C-shaped monomer structure contact the helical domain of the neighboring subunit, so both *Ll-*AbiK domains are involved in formation of the interfaces between protomers (Supplementary Figure S10A, B). In one tip, the N-terminus of the α1 helix (residues 1–5) from the fingers subdomain, α8 helix, and following loop (both from the palm subdomain) contact the α5′ helix and a loop between helices α4′ and α5′ from the helical domain of the neighboring protomer. In the other tip, the α2′ helix of the helical domain of one protomer contacts a loop between RT and helical domains (residues 286–294) from the neighboring protomer. Analysis of intersubunit contacts with PDBePISA tool indicated that the average buried surface area at the interfaces between two subunits within a trimer is 2642 Å^2^. The interactions between the two trimers of the *Ll-*AbiK hexamer are mediated by both the RT-like domain (through helices α9 and α11, α12/α13 intervening loop, helix α14 with the following loop, and strand β1) and helical domain (α8′/α9′ and α12′/α13′ intervening loops). The total buried surface area upon the interaction of two trimers is 8040 Å^2^. The arrangement of the subunits creates a spherical cavity in the central part of hexamer with a diameter of ∼40 Å.

Abi-P2 adopts a trimeric arrangement that is similar to *Ll-*AbiK (Figure 1C, D). The inter-protomer interactions are formed by α7 and α12 helices of the RT-like domain and α1′ helix of the helical domain, all located on one tip of one protomer and α2′, α4′, and α6′ helices of the helical domain from the second tip of the neighboring protomer (Supplementary Figure S10C, D). The average buried surface area at the interfaces between two subunits within a trimer is 1818 Å^2^. The asymmetric unit for the crystal structure contained six protein molecules that were arranged similarly to *Ll-*AbiK hexamers as two trimers stacked back-to-back. Therefore, Abi-P2 also has the potential to adopt a hexameric architecture. This is further supported by the results of the intersubunit contact analysis which estimated that the total buried surface area upon the interaction of two trimers of Abi-P2 forming a hexamer is 4100 Å^2^ (in HIV-1 RT, for example, which is known to be a very stable heterodimer, the total buried surface area at the interface between p66 and p51 subunits is 5330 Å^2^). However, in our experiments Abi-P2 at higher concentrations was only soluble in high ionic strength buffers which likely also inhibited hexamer formation. This would explain why in EM and MALS analyses only the trimeric form was observed.

When the *Ll-*AbiK hexamer and Abi-P2 trimer are superimposed based on the palm subdomain of one of their subunits, it is apparent that the overall triangular shape is common to both complexes (Figure 1A, C). The triangular cores of *Ll-*AbiK and Abi-P2 have almost identical dimensions, and the positions of the DNA-binding channels overlap. The C-terminal extensions of the helical domains of *Ll-*AbiK protrude from the triangular core, similar to propeller blades. The interfaces between subunits in the trimers are mostly formed by corresponding fragments of *Ll-*AbiK and Abi-P2. However, contacts between subunits that stabilize the *Ll-*AbiK hexamer involve structural elements that do not have counterparts in Abi-P2 (i.e. helix α11 and α12′/α13′ intervening loop). In summary, the cryo-EM and crystal structures of *Ll-*AbiK and Abi-P2 protomers reveal a bilobal architecture with RT-like and helical domains. The protomers are arranged as trimers and hexamers, which is a highly unusual feature for RT-like proteins.

### Structure of *Ll*-AbiK–DNA adduct reveals the mode of nucleic acid binding

The ability to catalyze protein-primed non-templated DNA synthesis has been reported for *Ll-*AbiK previously (7). The Abs_260_/Abs_280_ ratio of the purified protein sample was in the range of 1.05–1.15 indicating the presence of a nucleic acid. Based on the absorbance ratio we estimated that 10–12 nucleotides were present per *Ll*-AbiK protomer. We assumed that they represent a short DNA fragment that is covalently bound to *Ll-*AbiK and which was synthesized in the bacterial cell and co-purified with the protein. To confirm this, we performed a mass spectrometry analysis of purified *Ll-*AbiK. We observed a peak corresponding to the mass of free protein as well as a series of additional peaks corresponding to higher molecular weights which were regularly spaced at ∼320 Da intervals. This profile corresponded well with *Ll*-AbiK molecules with attached DNA fragments with the length of 9–17 nt (Supplementary Figure S11). Such DNA could indeed be observed in the crystallographic electron density map and in the cryo-EM density map. The fact that the DNA remained associated with the purified protein despite nuclease treatment of the sample at the first step of protein purification suggested that at least a part of the DNA chain was inaccessible to the nuclease. In the crystal structure of Abi-P2, no electron density was observed for the DNA, in agreement with the low Abs_260_/Abs_280_ ratio for this protein after purification.

**Figure 2.**
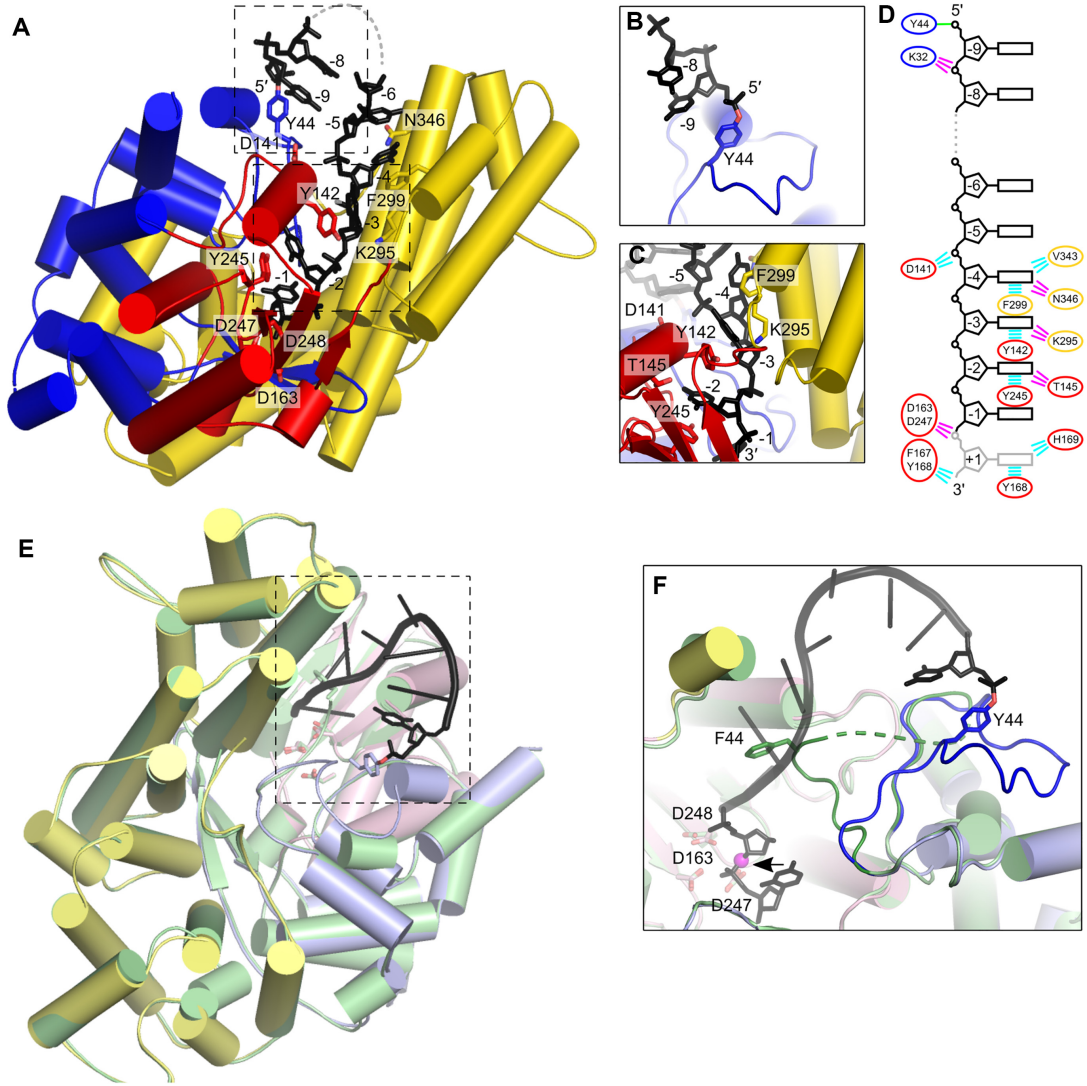
DNA binding by *Ll-*AbiK. (**A**) Cartoon representation of a single protomer of *Ll-*AbiK–DNA adduct showing the trajectory of the DNA product. Palm and fingers of the RT-like domain and helical domain are shown in red, blue, and yellow, respectively. DNA is shown as black sticks. Residues that are involved in DNA synthesis or engaged in contacts with the DNA are shown as sticks and labeled. Dashed boxes indicate the regions of the structure shown in panels (B) and (C). (**B**) Close-up of the priming site (different orientation). (**C**) Close-up of the active site (different orientation). (**D**) Schematic representation of protein-DNA contacts. The ovals are color-coded for the domains. The lines indicate interactions and are color-coded (polar interactions, magenta; van der Waals contacts, cyan; covalent linkage, green). Nucleotide at position +1 is only observed in the cryo-EM structure and in two subunits of the crystal structure. (**E**) Superposition of the crystal structure of wild-type *Ll-*AbiK and the cryo-EM structure of *Ll-*AbiK Y44F variant. The wild-type structure is colored pink for palm domain, light blue for fingers domain, and pale yellow for helical domain. DNA is shown as black sticks. AbiK Y44F structure is shown in pale green. Dashed box indicates the region of the structure shown in panel (F). (**F**) Close-up of the DNA-binding region (colored as in (E), different orientation). The mobile loop harboring the protein-priming residue is shown in dark blue and green for wild-type *Ll-*AbiK and the Y44F variant, respectively. The DNA is shown as black ladder with 5′ and 3′ terminal residues shown as sticks. Active site residues, the protein-priming tyrosine and phenylalanine substituting it are shown as sticks and labeled. The 3′ oxygen atom of the penultimate nucleotide, whose location indicates the approximate position of the hydroxyl group of Tyr44 which is required for the attachment of the first nucleotide, is shown as a magenta sphere and indicated with an arrow.

In the crystal structure of *Ll-*AbiK, eight to ten nucleotides of the DNA chain could be defined for individual protomers (Figure 2A). In all subunits, the DNA’s 5′ end is covalently attached to the hydroxyl group of Tyr44, which for the first time demonstrates that this tyrosine serves to prime DNA synthesis in *Ll*-AbiK (Figure 2B). Tyr44 is located in the middle of a long loop (residues 21–57) that essentially lacks secondary structure elements. We reasoned that conformational rearrangements of this region likely enable Tyr44 to enter the central channel between the polymerase and helical domains, thus allowing it to engage *in cis* with the active site and serve as a starting point for DNA synthesis. To test this assumption, we have prepared a *Ll-*AbiK variant in which Tyr44 is substituted with phenylalanine. This protein was unable to catalyze protein-primed DNA synthesis (see below), so we assumed that its structure would reveal the conformational state before nucleic acid polymerization starts. The structure of *Ll*-AbiK Y44F was determined at 2.7 Å resolution by cryo-EM and it is essentially identical to that of the wild-type enzyme except for a fragment of the 21–57 loop (Figure 2E). Residues 32–42 could not be traced in the *Ll*-AbiK Y44F structure implying a higher mobility and/or conformational heterogeneity of this region. The fragment spanning residues 43–49, which in the wild-type *Ll*-AbiK–DNA adduct structure runs parallel to the nucleic acid chain, adopts a different trajectory and runs toward the DNA-binding cleft (Figure 2F). As a result the location of the C-α of Tyr/Phe44 changes by 15 Å positioning this residue in the vicinity of the polymerase active site. This conformation very likely resembles the state in which the protein attaches the first nucleotide to the side chain of Tyr44. It should be noted, however, that in the structure the distance between the expected position of the protein-priming hydroxyl group is too large to be directly conducive to nucleotide transfer. The catalytic state would correspond to a conformation in which the hydroxyl group of Tyr44 moves ∼12 Å closer to the active site, so that it can perform a nucleophilic attack on the phosphate group of the incoming nucleotide. Perhaps such a conformation is a transient high-energy state that could not be captured in the structure. Nevertheless, the structure of Y44F variant reveals that a conformational change of the loop harboring the key tyrosine, which can position it for the protein priming at the active site, can be readily accommodated in the overall hexameric architecture of the enzyme.

In the wild-type *Ll*-AbiK–DNA adduct crystal structure, downstream from the first nucleotide that is attached to the tyrosine residue, the DNA strand makes a turn and continues through the channel between the polymerase and helical domain toward the active site, which is formed by Asp163, Asp247 and Asp248 and is located close to the center of the hexamer (Figure 2C). In this channel, nucleotides −2, −3 and −4 (numbering in 5′-3′ direction with nt −1 located near the active site) are stabilized by stacking interactions between nucleobases and aromatic side chains that line the sides of the channel (Tyr142, Tyr245 and Phe299) (Figure 2D, Supplementary Figure S7). Interactions are also formed with the phosphate backbone (mediated by Asp141) and with nucleobases (mediated by Tyr145, Lys295, and Asn346) (Figure 2D). Additionally, within the channel a part of the helical domain appears to stabilize the nascent DNA strand similarly to the template strand that stabilizes the DNA primer strand in RTs. In the crystallographic data a continuous electron density for the entire DNA chain is only observed in one of the subunits of the hexamer. For the other protomers, the electron density contains a gap after the second nucleotide. Similarly, in the cryo-EM reconstruction two separate fragments of the DNA product can be seen—three nucleotides of the 5′ portion of the DNA, including the terminal nucleotide that is covalently attached to the protein, and eight nucleotides of the 3′ fragment that are accommodated within the channel. This implies that the region connecting these fragments is mobile or the DNA length could vary among individual molecules.

In the cryo-EM structure of *Ll-*AbiK a clearly defined density of a 3′ nucleotide connected to the rest of the DNA is visible at the active site. The position of this nucleotide corresponds to the pre-translocation product state in which the last residue of the DNA occupies the position of the incoming nucleotide in substrate state. Similarly, in the crystal structure, electron densities are observed at the active sites of most of the protomers that likely correspond to a nucleotide in pre-translocation state. However, the poor quality of the density maps precluded model building as it indicated a large degree of disorder/mobility or, more likely, heterogeneity for this part of the structure.

Among the residues that were identified in *Ll*-AbiK as making important contacts with the DNA product, only Tyr245 has an obvious counterpart in the Abi-P2 structure (Tyr282). Other residues in *Ll-*AbiK are located in secondary structure elements that adopt different positions or are absent in Abi-P2. These elements may change their orientations upon DNA synthesis and come into contact with the covalently bound DNA, thereby stabilizing it. The identification of Abi-P2 residues that interact with the DNA will require the determination of a complex structure with nucleic acid.

In summary, the crystal and cryo-EM structures of the *Ll-*AbiK–DNA adduct reveal that DNA synthesis is primed by Tyr44 and shows the trajectory of the covalently bound DNA from the priming site to the active site that is located close to the center of the hexamer. The additional cryo-EM structure of Y44F variant shows the conformational rearrangements of the region that comprises the priming tyrosine which are necessary to initiate the DNA synthesis.

### Biochemical experiments confirm the importance of functional residues in *Ll*-AbiK and Abi-P2

**Figure 3.**
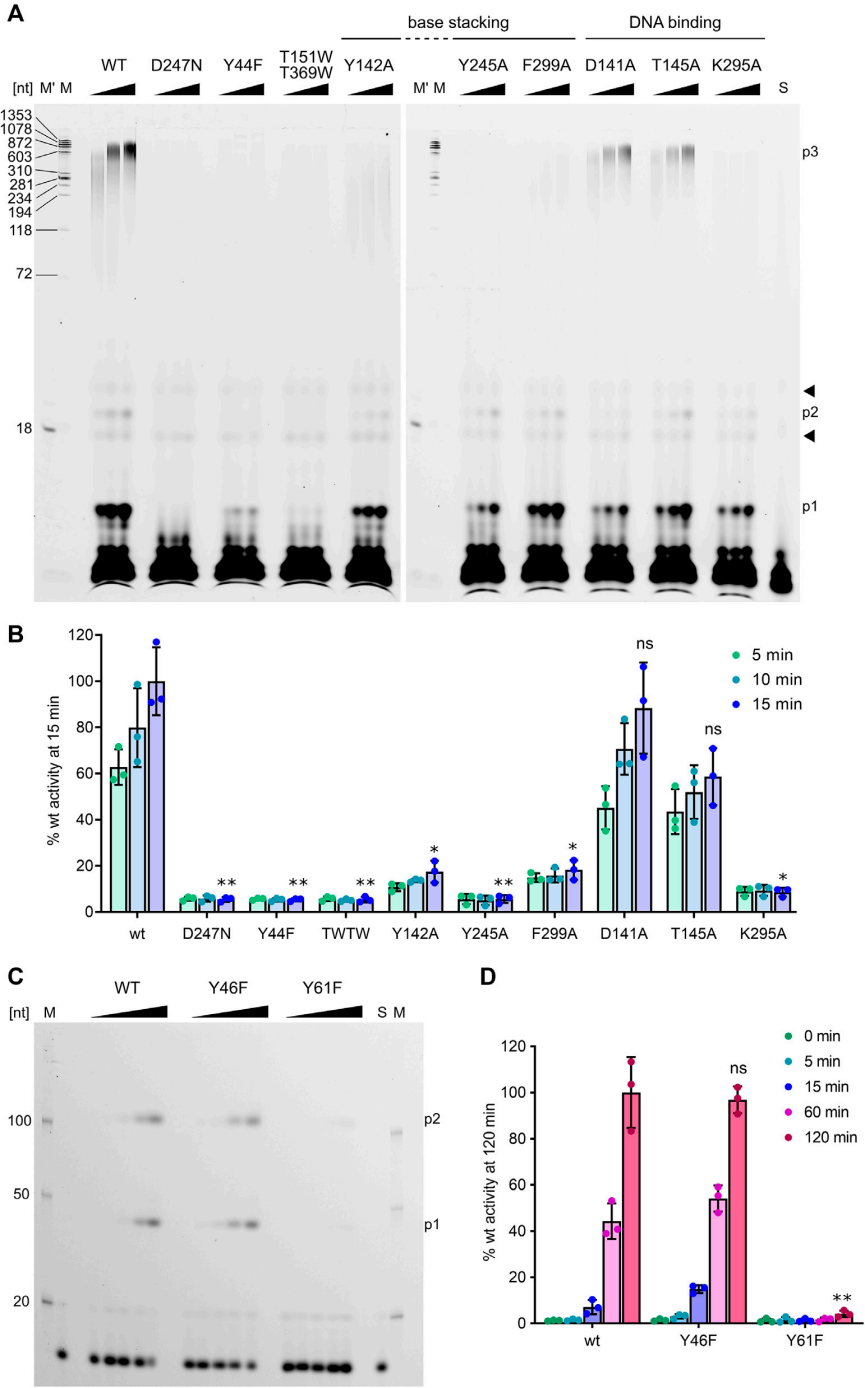
DNA synthesis by *Ll*-AbiK and Abi-P2 substitution variants. (**A**) Template-independent activity of *Ll-*AbiK variants in the presence of fluorescein-labeled dUTP, dNTP mix and 2 mM MgCl_2_ after 5, 10 and 15 min incubation at 37°C. Reaction products were analyzed on 10% TBE-urea polyacrylamide gels and visualized using fluorescent readout. The three main products are indicated as ‘p1’, ‘p2’ and ‘p3’. Arrowheads indicate the contaminants of fluorescein-dUTP that were used to correct for loading errors in quantitative analysis of results. M – DNA size ladder produced by enzymatic digestion of fluorescein-labeled ΦX174 DNA, M′ – 18 nt ssDNA labeled with fluorescein, S – lane with fluorescein-dUTP. (**B**) Densitometric quantification of long DNA products (marked ‘p3’ in A). The results were normalized with the amount of the product made by the wild-type enzyme in the last time-point expressed as 100%. Values obtained from three independent experiments are shown as circles, the bars represent means and error bars show the SD. Statistical significance of the difference between the value obtained for wild-type protein and a given variant for the last time point of each time-course was determined by the paired sample *t*-test and is indicated (*P < 0.05 and **P < 0.01, ns, not significant). (**C**) Activity of Abi-P2 variants in the presence of Texas Red-labeled dCTP, dNTP mix and 2 mM MgCl_2_ after 0, 5, 15, 60 and 120 min incubation at 37°C. Reaction products were analyzed on 10% TBE–urea polyacrylamide gels and visualized using fluorescent readout. The two products are marked as ‘p1’ and ‘p2’. S – lane with Texas Red-dCTP. M – DNA size markers labeled with Texas Red. (**D**) Quantification of the amount of DNA products (p1 + p2) from the assay shown in (C). Percentages of the fluorescent nucleotide incorporated into polymerization products from three independent experiments are plotted as circles. The results were normalized with the amount of the fluorescent nucleotide incorporated by the wild-type enzyme in the last time-point expressed as 100%. The bars represent the mean and error bars show the SD. Statistical significance between wild type and each variant by the paired sample *t*-test shown as in (B).

To verify the importance of the functional residues that were identified in the *Ll-*AbiK structures, we prepared a series of *Ll-*AbiK variants with one or two point substitutions of the residues that are involved in enzymatic activity, interaction with DNA, and oligomerization and tested their ability to synthesize DNA (Figure 3A). The reactions did not include any additional nucleic acids to serve as primers, however the elevated Abs_260_/Abs_280_ ratio suggested that some of the variants contained pre-synthesized ssDNA fragments that could further be extended. The main products of *Ll-*AbiK are long ssDNAs (up to 1000 nt), but two short ss products were also observed—approximately 10 and 20 nt long. The substitution of one of the catalytic residues (D247N) completely abolished protein activity. As expected, replacement of the protein-priming tyrosine with phenylalanine (Y44F) inactivated the enzyme, confirming the essential role of the hydroxyl group of Tyr44 in DNA synthesis. Importantly, as shown by the GF/MALS analysis, abolishing polymerase activity by the introduction of D274N substitution resulted in the protein forming almost exclusively monomers (Supplementary Figure S9). Similarly, cryo-EM analysis of the other inactive mutant, Y44F, showed that the majority of the particles were monomers and only a fraction of them, which was used for structure determination, corresponded to hexamers. This suggests that for *Ll-*AbiK, the presence of DNA stabilizes the protein conformation that is required for trimer formation.

The substitution of residues that are involved in DNA contacts affected polymerase activity to different degrees (Figure 3A, B). A reduction of activity was observed for variants with substitutions of the residues that formed stacking interactions with the bases of DNA. Y245A did not synthesize long DNA products, but small amounts of short products appeared at later time points. Y142A and F299A exhibited a severe reduction of catalytic activity, with the amount of long product after 15 min reaction reduced to 17.4% and 18.2% of the amount observed with the wild type enzyme, respectively. The D141A and T145A substitutions (substitutions of residues involved in van der Waals interaction with the phosphate backbone of nucleotide −4 and hydrogen bonding with nucleobase of nucleotide −2, respectively) affected *Ll-*AbiK activity moderately and this reduction was not statistically significant. Finally, alanine substitution of Lys295, which forms a hydrogen bond with a nucleobase of nucleotide −4, resulted in a reduction of DNA polymerase activity to 8.6% of the activity of the wild-type protein.

We also assayed the activity of a *Ll-*AbiK variant that harbored a double substitution of residues that are involved in contacts that stabilize the *Ll-*AbiK trimer. We introduced substitutions with tryptophans whose bulky side chains should disrupt the extensive intersubunit interface (T151W/T369W). Fourier Transform Infrared Spectroscopy measurement demonstrated that introduction of these substitutions did not affect the secondary structure content of the protein (Supplementary Figure S12). This variant was analyzed by GF/MALS experiments and was shown to exist in monomeric form (measured MW of 71.6 kDa versus theoretical MW of the monomer of 71.5 kDa) (Supplementary Figure S9). The double mutant did not display any polymerase activity, indicating that trimer and/or hexamer formation is required for DNA synthesis by *Ll-*AbiK. In this context, it would be interesting to study *Ll*-AbiK trimers, however we were unable to produce variants in which only hexamer but not trimer formation would be perturbed.

We next tested the DNA polymerase activity of Abi-P2. Two distinct DNA products were observed—one approximately 40 nt long and the other longer than 100 nt (Figure 3C). The absence of DNA in the structure precluded identification of the tyrosine residue that is responsible for polymerization initiation. Two tyrosine residues (Tyr46 and Tyr61) are present in the region of Abi-P2 that corresponds to the region of AbiK that comprises its priming tyrosine. We individually substituted these two tyrosines with phenylalanine. The Y46F variant retained wild-type levels of activity, whereas the Y61F variant did not polymerize DNA, showing that the hydroxyl group of the latter residue is involved in DNA synthesis priming (Figure 3C, D). This is in agreement with the fact, that although the position of Tyr61 does not strictly correspond to that of *Ll*-AbiK Tyr44, both of these residues are located on the loop connecting helices α2 and α3 of the fingers subdomains. It should be noted that in this study only the *in vitro* activity was assessed and for the *in vivo* activity the involvement of host factors needs to be considered. Potential limits of the assay, or potential lability of the covalent bond between DNA and tyrosine residue also need to be taken into account. Moreover, the activity of *Ll*-AbiK and Abi-P2 in our *in vitro* assays cannot be directly compared. The assays for the two proteins were performed at different concentrations of the enzymes and nucleotides. The reactions for *Ll*-AbiK had a 2.5-fold higher concentration of fluorescently labeled dNTP and the fluorescent label was fluorescein which has much higher fluorescence quantum yield than Texas Red used in Abi-P2 reactions.

In summary, the biochemical experiments confirmed the involvement of Abi-P2 Tyr61 in DNA priming and revealed the role of several *Ll-*AbiK residues in the central channel in stabilizing the nascent DNA product. Furthermore, our results showed that for *Ll-*AbiK, the presence of the DNA plays a role in trimer/hexamer stabilization, and, reciprocally, *Ll-*AbiK multimerization is required for DNA polymerase activity.

### Comparison with other RTs reveals common and divergent structural elements

**Figure 4.**
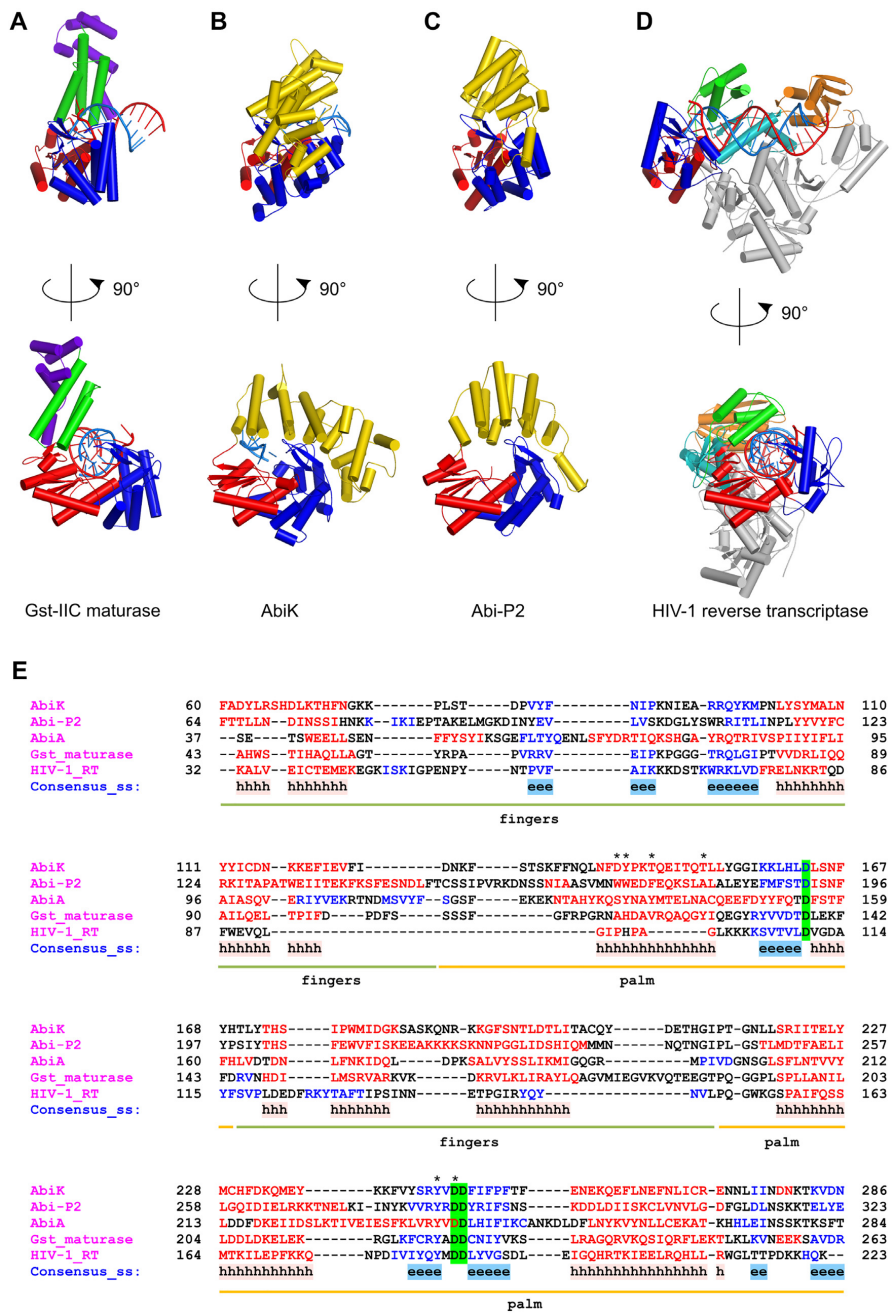
Structural comparison of Abi proteins with group II intron maturase and HIV-1 reverse transcriptase. Structures of *G. stearothermophilus* maturase (PDB ID: 6AR1(38)) (**A**), *Ll-*AbiK (**B**), Abi-P2 (**C**) and HIV-1 RT (PDB ID: 4PQU(40)) (**D**) are shown in two orientations. Domains are color-coded: palm, red; fingers, blue; helical domain, yellow; thumb, green; connection domain, teal; RNase H, orange; DNA-binding domain, violet. The p51 subunit of HIV-1 RT is shown in gray. DNA and RNA strands in substrates are shown as cartoon and colored blue and red, respectively. (**E**) Sequence alignment of fingers/palm portion of *Ll*-AbiK and Abi-P2 polymerases with corresponding regions of *L. lactis* AbiA, *G. stearothermophilus* maturase and HIV-1 RT (output from PROMALS3D (41)). The alignment was truncated to show only the regions that can be reliably superimposed structurally. Residue ranges corresponding to fingers and palm domains are marked below the alignment with green and yellow lines, respectively. Secondary structure elements are indicated with colors – α-helices in red, β-strands in blue. Active site residues are highlighted in green. Positions at which substitutions were introduced in *Ll*-AbiK are indicated with asterisks above the alignment.

*Ll-*AbiK and Abi-P2 are classified as members of the RT superfamily, based on sequence similarity. Their sequences contain four conserved RT motifs that are located in the palm and fingers subdomains of the polymerase (28). As expected, similarities to RTs can also be observed in the structures. We compared the structures and the sequences of *Ll-*AbiK–DNA adduct and Abi-P2 to two representative structures of RTs in complex with their RNA/DNA substrates: group II intron maturase from *G. stearothermophilus* (Gst-IIC RT) and human immunodeficiency virus 1 (HIV-1) RT (Figure 4). In these comparisons, the highest structural similarity is observed for the palm subdomain, with some important differences. For example, *Ll-*AbiK lacks the 2–3 short β-strands that form a small β-sheet that is present in the C-terminal region of the palm in maturase and HIV-1 RT. This region in HIV-1 RT and maturase is located adjacent to the polymerase active site and plays important roles in positioning of the thumb subdomain and stabilization of the 3′ terminus of the primer in the double-helical nucleic acid substrate. Abi RTs lack the thumb subdomain, and the part of their structure that corresponds to the small β-sheet is involved in trimer formation. Moreover, Abi proteins bind single-stranded nucleic acids, and the DNA strand has a different trajectory compared with DNA primers in typical RTs. Therefore, the lack of the small β-sheet is an adaptation to different structural architecture and type of substrate of Abi RTs.

The fingers subdomain of *Ll-*AbiK is larger than in the other two RTs and comprises nine helices compared with five and three helices of the maturase and HIV-1 RT, respectively. Among the most conserved features of the fingers subdomain is the β-hairpin that contains residues that have been identified as key determinants for dNTP misinsertion and mispair extension in HIV-1 RT. The best studied of these residues, Lys65 (29), has its counterpart in *Ll-*AbiK (Lys90), but its role is unclear given the untemplated mode of DNA synthesis by *Ll-*AbiK. Another important hairpin residue is an arginine (Arg72 in HIV-1 RT) which stabilizes the incoming nucleotide and is also conserved both in *Ll-*AbiK (Arg96) and in the maturase (Arg75) (30). The structural environment of the β-hairpin is different in the three structures. In *G. stearothermophilus* maturase, the β-hairpin interacts only with RNA, whereas in HIV-1 RT it is positioned between the template RNA and a long helix of the fingers subdomain. In *Ll-*AbiK, the β-hairpin runs along the bottom edge of the helical domain and forms one of the key contact points between polymerase and helical domains.

Characteristic elements/motifs of the polymerase domain of group II intron maturases, which are absent from retroviral RTs, are N-terminal extension (NTE), RT0, RT2a and RT3a (31). Most of these elements are also found in Abi polymerases, however they exhibit different degrees of structural similarity to their maturase counterparts (Supplementary Figure S13).

As mentioned above, the polymerase domain of *Ll-*AbiK does not contain a thumb subdomain. Instead, its position in space is occupied by the first three helices of the helical domain of *Ll-*AbiK. The recent structures of the Cas6-RT-Cas1 fusion protein from *Thiomicrospira* showed that this protein also lacks the thumb subdomain (32). Therefore, loss or replacement of the thumb subdomain can occur to accommodate various functions of the polymerase domain.

Unlike in *bona fide* RTs that synthesize DNA using an RNA or DNA template and bind double-stranded helical nucleic acid, the DNA that interacts with *Ll-*AbiK is single-stranded and adopts an extended conformation. Consequently, the enzyme-nucleic acid interactions are different. In *Ll-*AbiK, the 3′ terminal fragment of the DNA strand runs close to the α8 helix of the palm subdomain and forms very few interactions with the fingers subdomain. However, the position of the 3′ terminal nucleotide of the pre-translocation state captured in the *Ll-*AbiK-DNA structure is identical to the position of the incoming nucleotide in RNA/DNA complexes of the maturase and HIV-1 RT, reflecting the conservation of active site architecture. In *G. stearothermophilus* maturase and HIV-1 RT, the RNA/DNA substrate is bound in a cleft that is formed by the thumb-palm-fingers. In *Ll-*AbiK, the space between the helical domain and fingers subdomain is much smaller and narrower, thus precluding accommodation of the double-helical nucleic acid. In summary, Abi polymerases share palm and fingers subdomains with other RT proteins but lack the thumb subdomain. The unique feature of Abi polymerases is the helical domain that stabilizes the single-stranded DNA product and participates in trimer formation.

## DISCUSSION

Here, we describe the structures of two bacterial polymerases that are involved in abortive infection anti-phage mechanism. These are unique enzymes that are capable of autonomous protein priming combined with the template-independent synthesis of DNA. We report crystal and cryo-EM structures of representatives of two distantly related Abi DNA polymerase families: AbiK and Abi-P2. The structures reveal an intriguing and unique mechanism of covalent attachment of the DNA to a tyrosine residue. The helical domain that is unique for Abi proteins serves to stabilize the nascent DNA chain in which it replaces the template strand. *Ll-*AbiK and Abi-P2 form hexamers and trimers, respectively. This is an unexpected feature of RT-related proteins. The adoption of this oligomeric architecture is possible because of the presence of the helical domain that plays a key role in trimer/hexamer contacts. In *Ll-*AbiK, the presence of the DNA strand stabilizes trimer formation and the trimerization is in turn required for DNA synthesis. Therefore, there is an intimate link between trimer formation and unique catalytic activity of the enzyme. This implies that trimerization evolved to achieve the distinctive feature of Abi proteins, namely the ability to synthesize DNA in a template-independent manner. For Abi-P2, the relationship between trimerization and DNA polymerization is different, as the protein is a stable trimer in the absence of the DNA.

The phenomenon of protein-primed DNA synthesis has been described for several DNA polymerases. The best studied examples include the polymerases of the *Bacillus* phi29 bacteriophage, adenoviruses and hepadnaviruses. The only other protein-priming DNA polymerase for which structural data is available is the phi29 enzyme. Unlike Abi polymerases which represent the RT-family of DNA polymerases, the phi29 enzyme belongs to the B-family of DNA polymerases and thus has a very different architecture. Also, the protein-priming mechanism is different between these enzymes. In phi29, the serine residue that is used for priming of DNA synthesis is provided *in trans* by an accessory TP protein, whereas in Abi polymerases the priming occurs *in cis*. *In cis* protein-priming is also used by another RT-family DNA polymerase—the HBV P protein. However, no structural information is currently available for this enzyme. As a result, the structures presented in this study provide the first structural information about *in cis* protein-priming in RT-family of DNA polymerases.

The ability to catalyze addition of untemplated nucleotides has been observed for some DNA polymerases, including reverse transcriptases (33). For example, Moloney murine leukemia virus RT was shown to add up to seven nucleotides to blunt DNA ends resulting in 3′ overhangs (34). However, due to the architecture of their central channel, *Ll*-AbiK and Abi-P2 cannot accommodate and elongate double-stranded DNA. Moreover, the Abi polymerases are unique in their ability to synthesize much longer single-stranded DNA with tens (Abi-P2) to hundreds (*Ll*-AbiK) of nucleotides, as observed in *in vitro* reactions.

The *in vitro* activity assay demonstrated that even though wild-type *Ll*-AbiK and some of its variants already contained covalently attached short ssDNA strands, incubation with a dNTP mix resulted in longer DNA products. One explanation for this is that only short DNA fragments are synthesized in the bacterial cells and their elongation is possible thanks to favorable reaction conditions *in vitro* and lack of potential inhibitory factors that could be present in the bacterial cell. This would be in agreement with the observation that although the gene encoding *Ll*-AbiK is constitutively expressed, the enzyme does not exert its toxic effect until the phage infection occurs (6). An alternative explanation would be that *Ll*-AbiK produces longer DNA strands already in the cell, but during protein purification they are degraded by viscolase that is included in the lysis buffer. This leaves intact only short covalently attached DNA fragments that are not accessible to the nuclease and which can be further extended in our *in vitro* experiments.

The use of template-independent DNA polymerization and protein priming make AbiK and Abi-P2 completely autonomous—they can make long stretches of random-sequence DNA from scratch. Key outstanding questions are how this unique activity is regulated and triggered and how it induces bacterial cell death. The insensitivity of certain phages to AbiK has been linked to mutations of genes that result in a single amino acid substitution in protein products, termed Sak (for sensitivity to AbiK) (8). Some Sak proteins have been shown to be related to the single-strand annealing proteins RAD52 and Erf that are involved in homologous recombination (35–37). This implies a link between DNA recombination and AbiK-induced cell death. The massive DNA synthesis may also deplete dNTP pools in the cell and/or create crowding/entanglement in the cytoplasm. Future *in vivo* studies should investigate this issue.

In conclusion, our data provide a comprehensive view of the structure and molecular mechanism of unique Abi polymerases and explain their ability to autonomously polymerize DNA. They also provide a basis for future biotechnological applications. Our structures, together with structures of group II intron maturases and CRISPR-associated RT (32,38,39), also fill important gaps in our knowledge of very diverse bacterial RTs.

## DATA AVAILABILITY

Atomic model of *Ll-*AbiK and the corresponding cryo-EM reconstruction have been deposited with the Protein Data Bank (PDB) and EMDB under accession numbers: 7R06 and EMD-14220, respectively. Atomic model of *Ll-*AbiK Y44F and the corresponding cryo-EM reconstruction have been deposited with the Protein Data Bank (PDB) and EMDB under accession numbers: 7Z0Z and EMD-14435, respectively. Atomic coordinates and structure factors for the reported crystal structures of *Ll-*AbiK–DNA adduct and Abi-P2 have been deposited with the Protein Data Bank (PDB) under accession numbers 7R07, 7R08, respectively.

## Supplementary Material

gkac772_Supplemental_File
