# Supplementary material for: Mechanism of protein-primed template-independent DNA synthesis by Abi polymerases
Source: Nucleic Acids Res. 2022 Sep 15;50(17):10026–40. doi: 10.1093/nar/gkac772 (PMC9508834; doi:10.1093/nar/gkac772)
Supplement: gkac772_Supplemental_File [file gkac772_supplemental_file.pdf]

Mechanism of protein-primed template-independent DNA synthesis by Abi polymerases

Małgorzata Figiel, Marta Gapińska, Mariusz Czarnocki-Cieciura, Weronika Zajko, Małgorzata Sroka, Krzysztof Skowronek, and Marcin Nowotny

SUPPLEMENTARY INFORMATION

SUPPLEMENTARY FIGURES AND TABLES

Supplementary Table S1. EM data collection and processing

Supplementary Table S2. Refinement and validation statistics for cryo-EM models

Supplementary Table S3. Crystallography data collection and refinement statistics

Supplementary Figure S1. *Ll*-AbiK cryo-EM data processing

Supplementary Figure S2. Final steps of *Ll*-AbiK cryo-EM data processing

Supplementary Figure S3. Quality of *Ll*-AbiK cryo-EM map

Supplementary Figure S4. *Ll*-AbiK Y44F cryo-EM data processing

Supplementary Figure S5. Final steps of *Ll*-AbiK Y44F cryo-EM data processing

Supplementary Figure S6. Electron density maps for *Ll*-AbiK

Supplementary Figure S7. Superposition of crystal and cryo-EM structures of *Ll*-AbiK

Supplementary Figure S8. Abi-P2 cryo-EM data processing

Supplementary Figure S9. Gel filtration/multi-angle light scattering (GF/MALS) analysis of *Ll*-AbiK variants and Abi-P2

Supplementary Figure S10. Interaction between protomers within *Ll*-AbiK and Abi-P2 trimers

Supplementary Figure S11. Mass-spectrometry analysis of *Ll*-AbiK variants.

Supplementary Figure S12. Secondary structure content of wild-type *Ll*-AbiK and T151W/T369W variant.

Supplementary Figure S13. Structural motifs shared between maturase, *Ll*-AbiK and Abi-P2

Supplementary Table S1. EM data collection and processing.

	Ll-AbiK	*Ll*-AbiK Y44F	Abi-P2
Microscope	Glacios	Titan Krios G3i	Titan Krios G3i
Camera	Falcon 3EC	Gatan K3	Gatan K3
Energy filter	none	Gatan BioQuantum with 20 eV slit	Gatan BioQuantum with 20 eV slit
Voltage (kV)	200	300	300
Magnification	150 000x	150 000x	105 000x
Electron exposure (e/Å^2)	40	60	61
Defocus range (μm)	-1.6 to -0.6	-2.5 to -1.5	-2.0 to -1.0
Pixel size (Å)	0.95	0.82	0.86
Stage tilt (deg)	0	20 and 30	40, 50, and 60
Initial particle images (no.)	748 962	2 306 347	614 818
Final particle images (no.)	227 461	50 340	75 552
Symmetry imposed	D3	D3	C3
Resolution at 0.143 FSC	2.27	2.68	3.86
Resolution range at atom	2.13–4.92*	2.43-4.23*	N/A
Map sharpening B factor (Å^2)	-95	-75	-30
EMDB accession code	EMD-14420	EMD-14435	-

* range of the local resolution map values at atom positions

Supplementary Table S2. Refinement and validation statistics for cryo-EM models.

	Ll-AbiK	*Ll*-AbiK Y44F
PDB accession code	7R06	7Z0Z
Non-hydrogen atoms	31320	29466
Protein residues	3588	3510
Nucleotides	66	0
Bonds (RMSD): Length (Å)	0.006	0.006
Bonds (RMSD): Angles (°)	0.828	0.719
Validation: Rotamer outliers (%)	5.7	0.54
Ramachandran plot (%):<br>Outliers<br>Allowed<br>Favored	 0.17<br>1.18<br>98.65	 0.17<br>1.87<br>97.96

Supplementary Table S3. Crystallographic data collection and refinement statistics.

	Ll-AbiK	Abi-P2
**Data collection**		
Space group	$P6_2$ 2 2	$P2_1$
Cell dimensions		
a, b, c (Å)	201.0, 201.0, 570.7	134.3, 88.4, 161.1
α, β, γ (°)	90, 90, 120	90, 103.2, 90
Resolution (Å)	49.6 – 3.1 (3.29 – 3.1)	47.3 – 3.1 (3.28 – 3.1)
R_{merge}	0.197 (4.78)	0.266 (9.52)
$I/\sigma I$	14.18 (0.81)	12.22 (1.14)
$CC_{1/2}$	99.9 (34.5)	99.8 (65.7)
Completeness (%)	99.6 (99.1)	99.3 (97.5)
Multiplicity	21.6 (21.3)	4.8 (4.8)
**Refinement statistics**		
Resolution (Å)	49.6 – 3.1	47.9 – 3.1
No. of reflections	122650	66904
R_{work} (%)	18.9	22.0
R_{free} (%)	23.2	27.4
No. of atoms	30644	22211
macromolecules	30619	22211
ligands/ions	9	-
water	16	-
B factors (Å)		
macromolecules	116.9	106.3
ligands	132.3	-
Root mean square deviations		
Bond lengths (Å)	0.002	0.002
Bond angles (°)	0.51	0.43

Statistics for the highest-resolution shell are shown in parentheses.

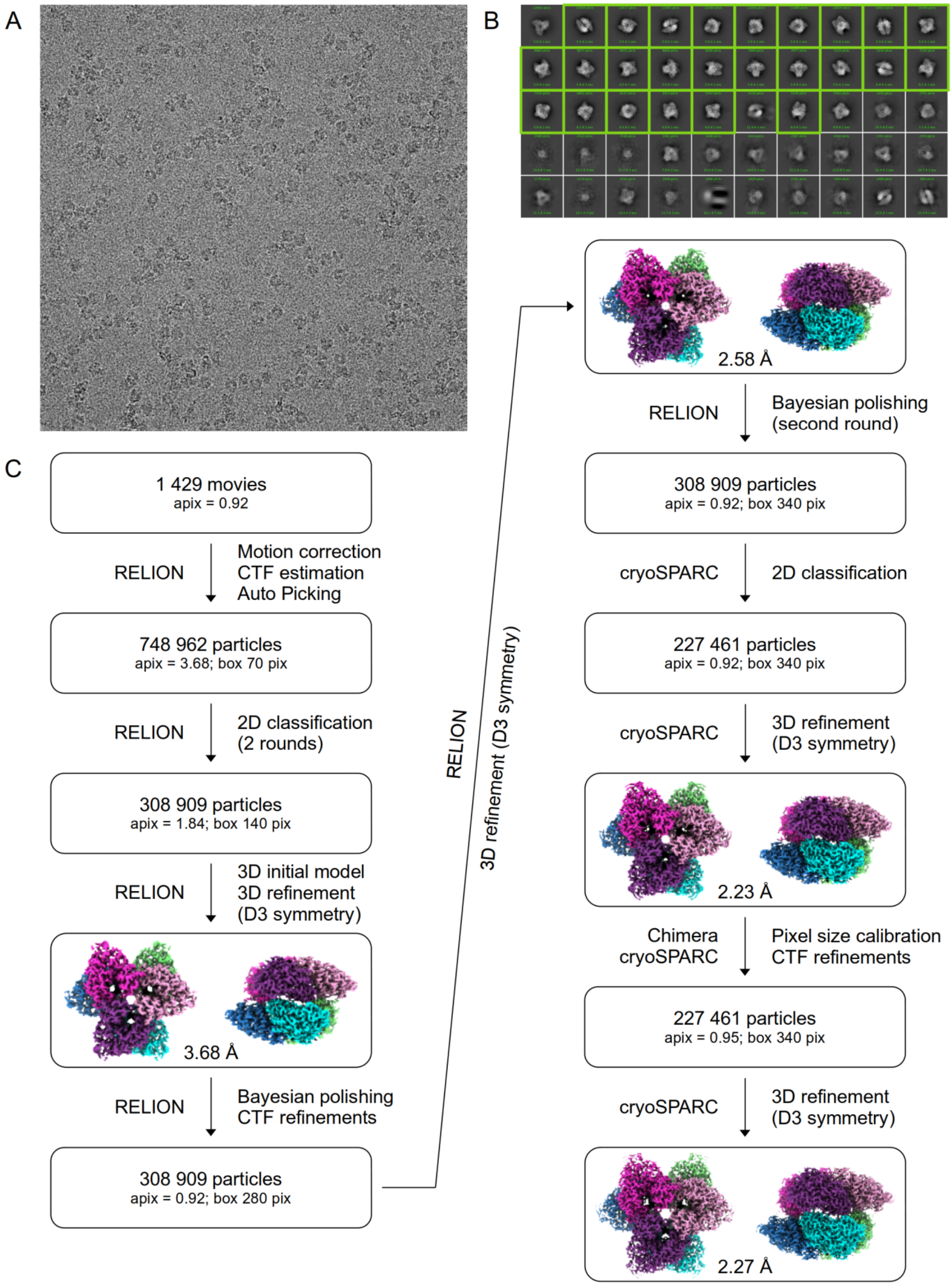

Supplementary Figure S1. ***Ll*-AbiK cryo-EM data processing.** (**A**) Representative micrograph. (**B**) Class averages from the last round of 2D classification in cryoSPARC. Green boxes indicate 2D class averages with selected particles. (**C**) Three-dimensional reconstruction pipeline.

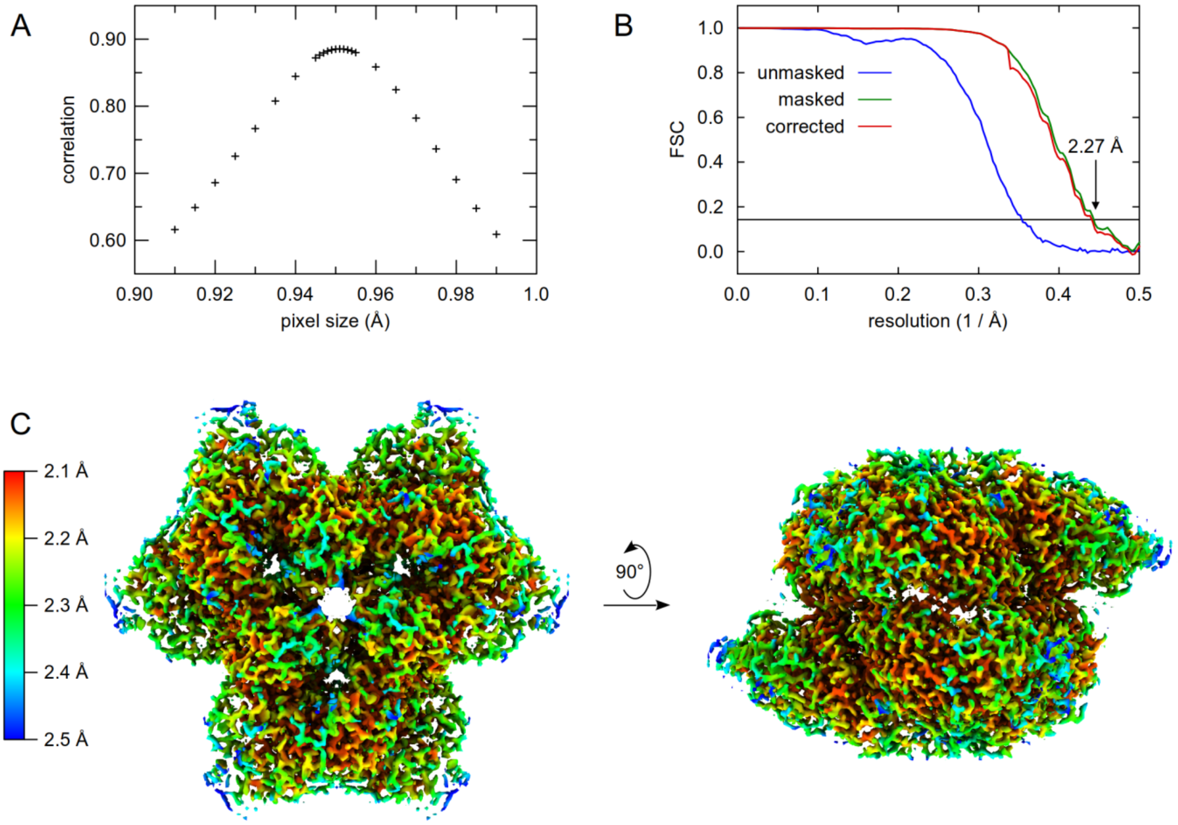

Supplementary Figure S2. **Final steps of *Ll*-AbiK cryo-EM data processing.** (**A**) Pixel size calibration. Correlation between EM map with altered pixel size and crystallographic model calculated with UCSF Chimera. (**B**) Gold-standard Fourier Shell Correlation (FSC) curve between two half maps for the final reconstruction. (**C**) Local resolution calculated from half maps in cryoSPARC. All maps were rendered with UCSF ChimeraX(42).

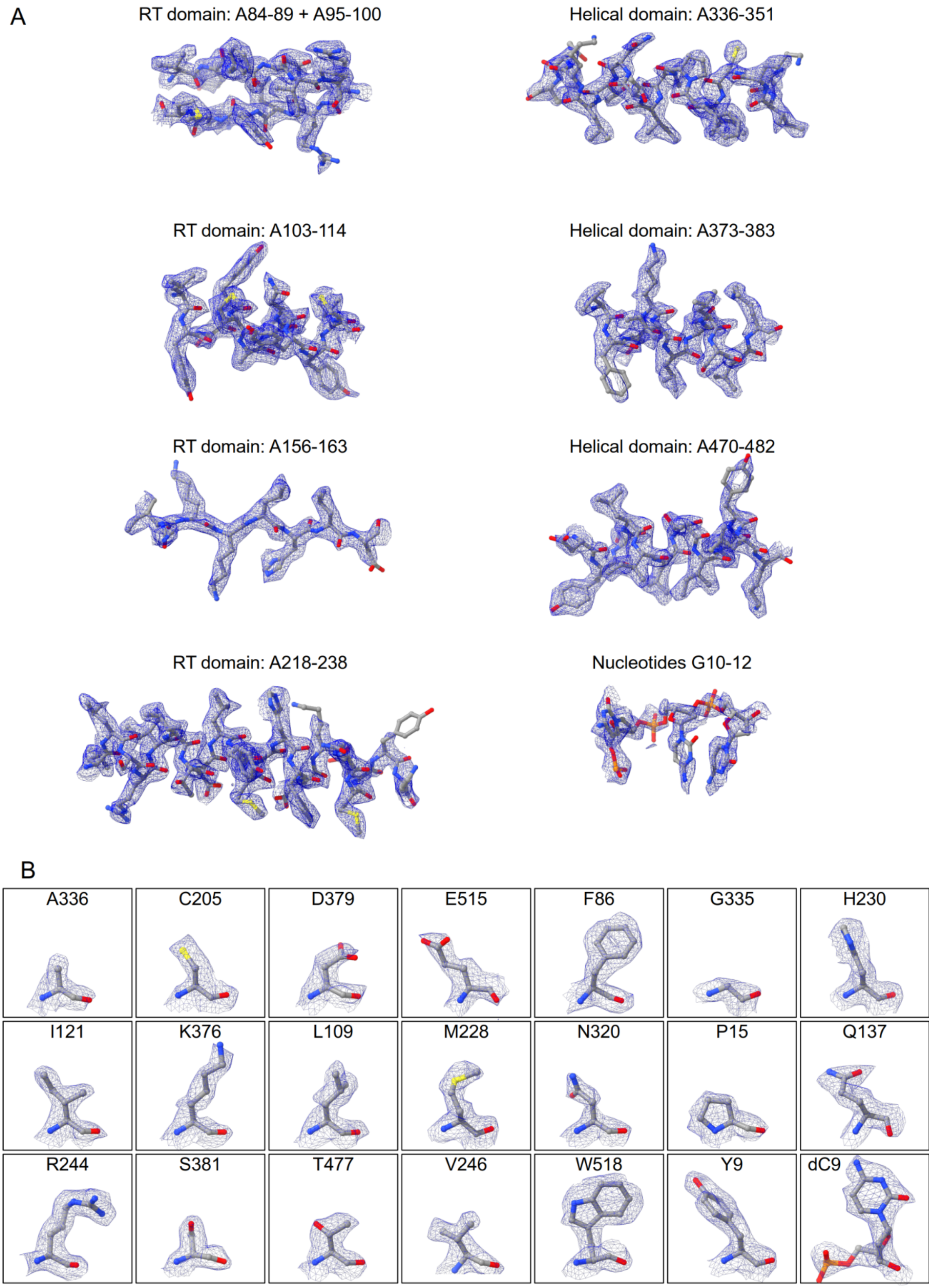

Supplementary Figure S3. **Quality of *Ll*-AbiK cryo-EM map.** (**A**) Selected secondary structures rendered with a threshold of 2.5. (**B**) Selected side chains rendered with a threshold of 2.5 (20 amino acids) or 1.2 (nucleotide – cytosine).

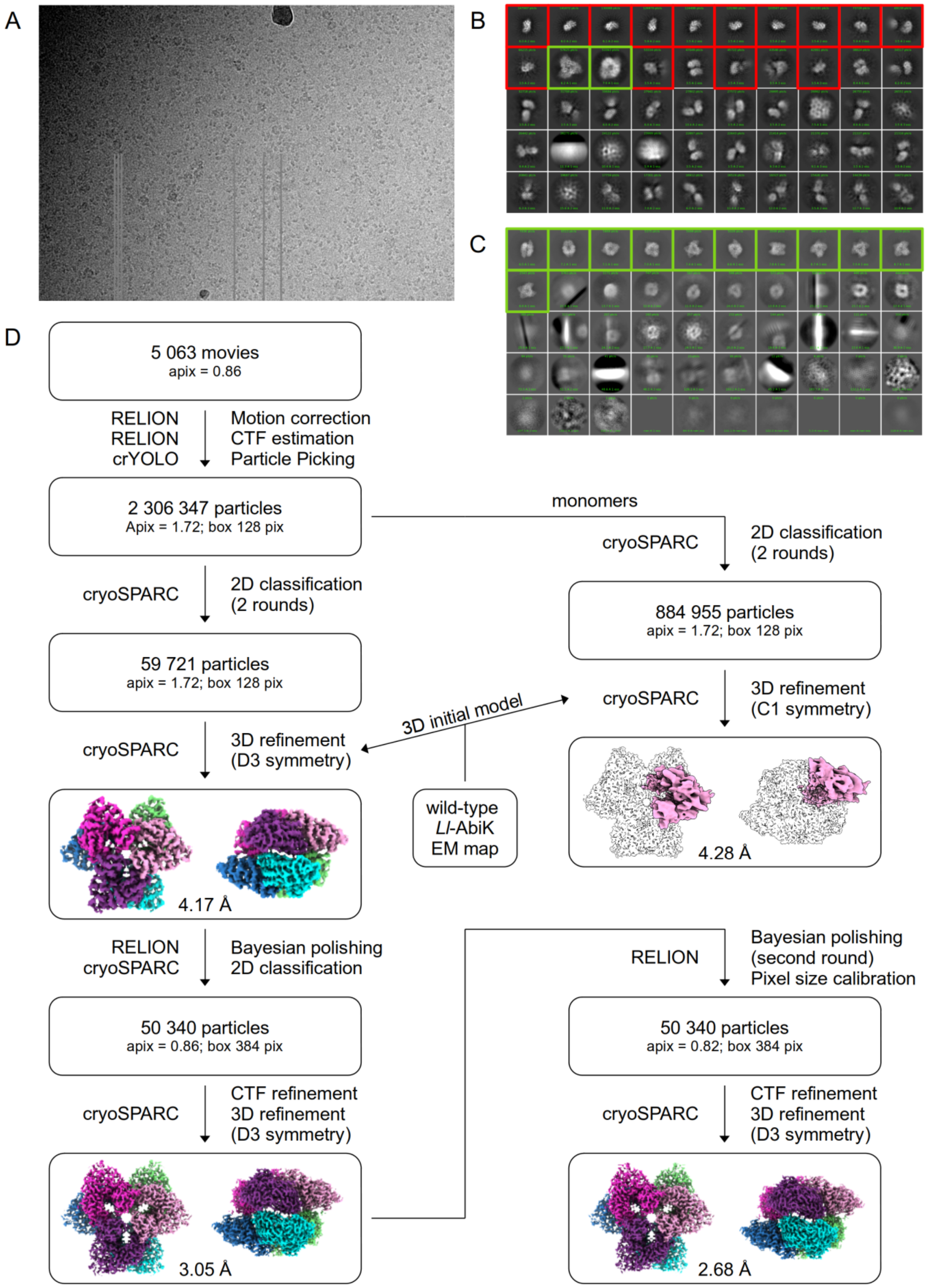

Supplementary Figure S4. ***Ll*-AbiK Y44F cryo-EM data processing.** (**A**) Representative micrograph. (**B**) Class averages from the first round of 2D classification in cryoSPARC. Red and green boxes indicate 2D class averages for monomeric and trimeric/hexameric particles, respectively. (**C**) Class averages from the last round of 2D classification of hexameric particles in cryoSPARC. Green boxes indicate 2D class averages with selected hexameric particles. (**D**) Three-dimensional reconstruction pipeline. An additional branch representing the processing of *Ll*-AbiK Y44F monomers has been included. The low quality reconstruction of the monomer is shown in alignment with the final hexameric reconstruction shown as white contour.

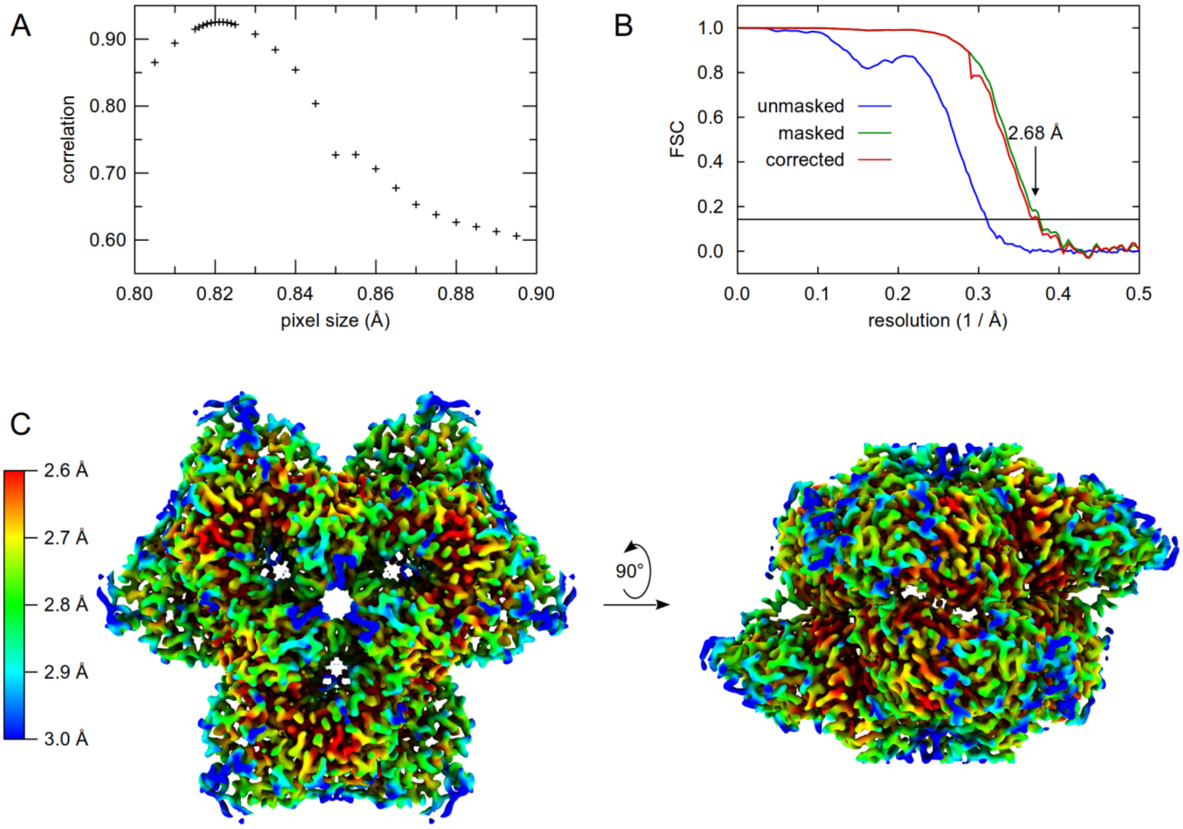

Supplementary Figure S5. **Final steps of *Ll*-AbiK Y44F cryo-EM data processing.** (**A**) Pixel size calibration. (**B**) Gold-standard Fourier Shell Correlation (FSC) curve between two half maps for the final hexamer reconstruction. (**C**) Local resolution calculated from half maps in cryoSPARC. All maps were rendered with UCSF ChimeraX(42).

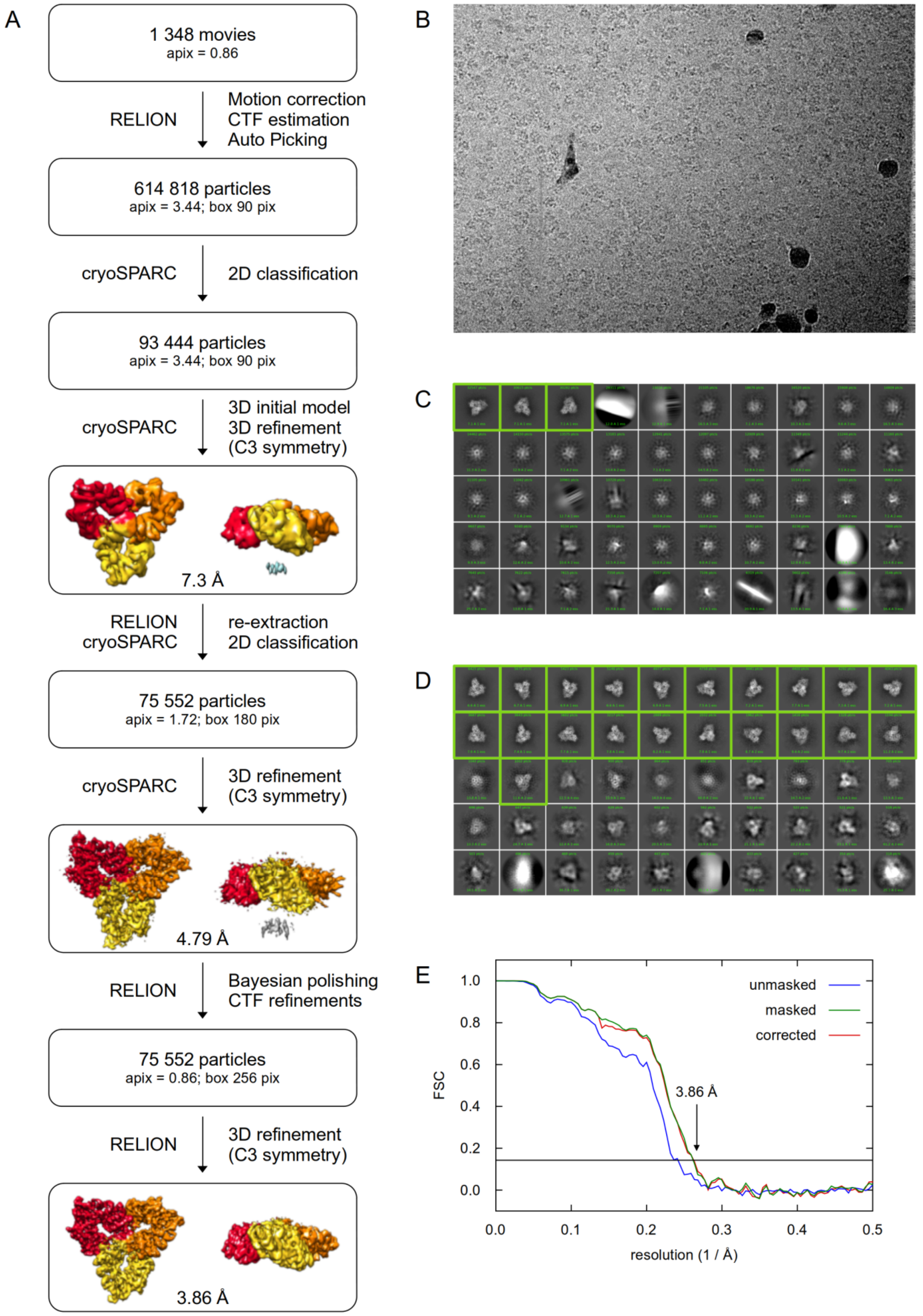

Supplementary Figure S6. **Abi-P2 cryo-EM data processing.** (**A**) Three-dimensional reconstruction pipeline. (**B**) Representative micrograph. (**C**, **D**) Class averages from the first and second round of 2D classification in RELION. Green boxes indicate 2D class averages with selected particles. (**E**) Gold-standard Fourier Shell Correlation (FSC) curve between two half maps for the final reconstruction. All maps were rendered with UCSF ChimeraX(42).

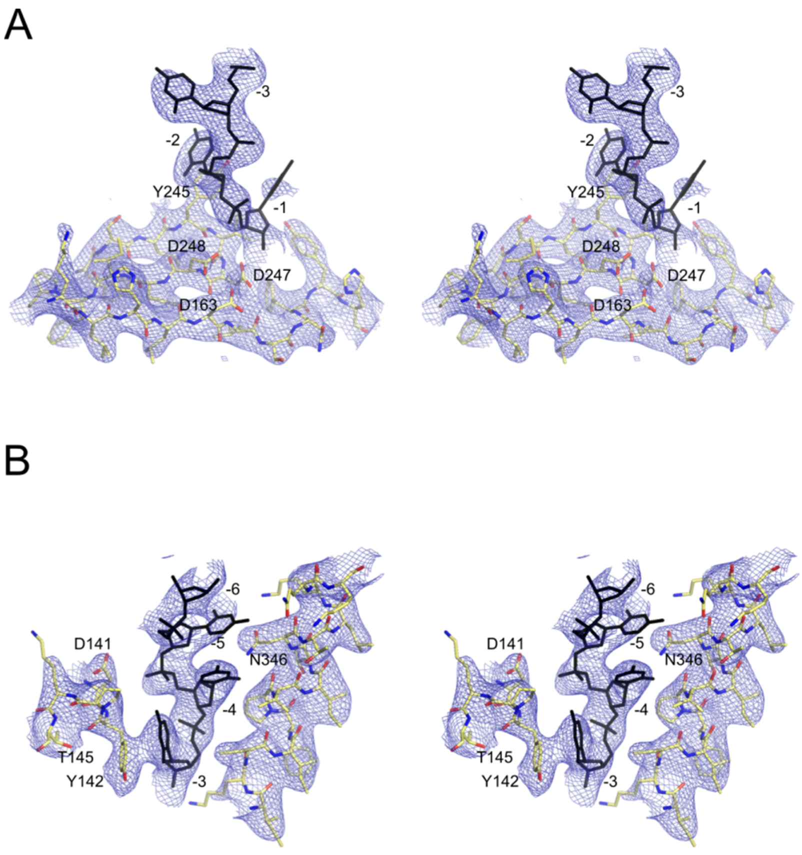

Supplementary Figure S7. **Electron density maps for *Ll*-AbiK.** (**A**, **B**) Stereo view of simulated annealing omit electron density maps contoured at 2.0 σ overlaid on fragments of the structure around the active site (A) and around the DNA in the central channel (B).

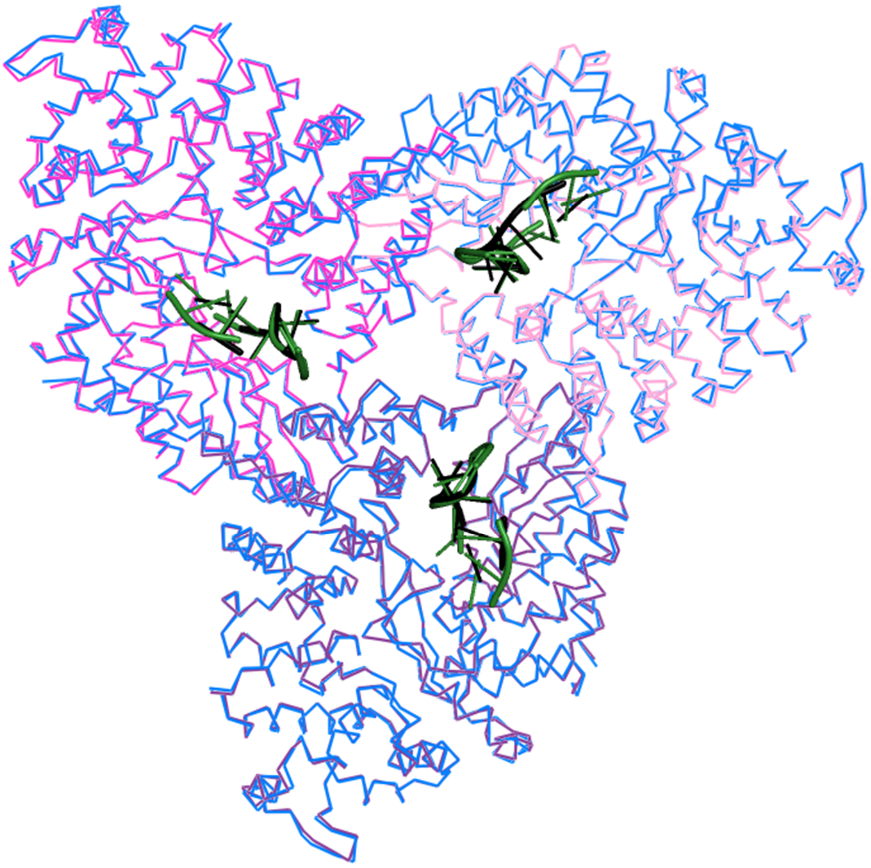

Supplementary Figure S8. **Superposition of crystal and cryo-EM structures of *Ll*-AbiK.** Structures of *Ll*-AbiK trimers determined by X-ray crystallography and cryo-EM are shown. For the crystal structure, protein chains are shown as wire and colored pink, magenta and violet, DNA is shown as black cartoon. For the cryo-EM structure, protein is shown as blue wire and DNA as green cartoon.

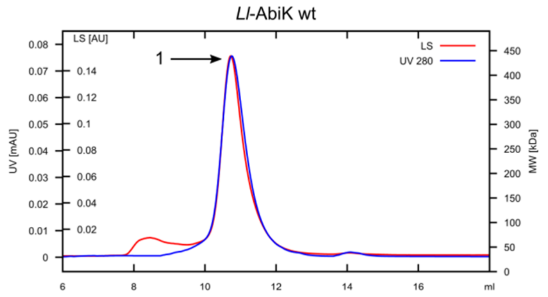

1. 413.7 kDa (±0.34%)

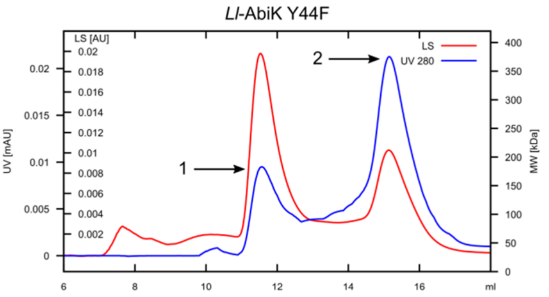

1. 257.0 kDa (±0.94%)
2. 62.0 kDa (±0.99%)

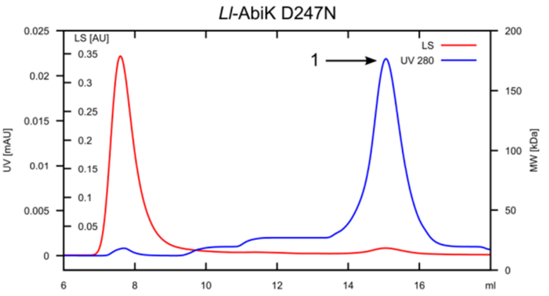

1. 93.0 kDa (±2.6%)

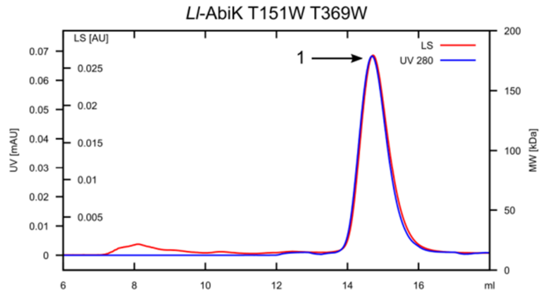

1. 71.6 kDa (±0.73%)

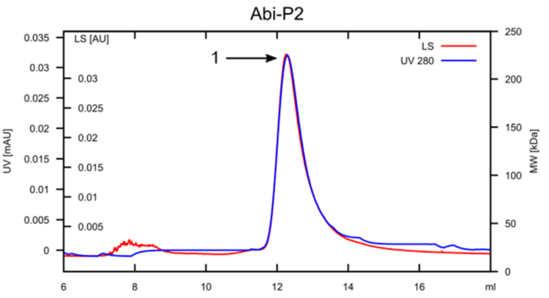

1. 182.6 kDa (±0.33%)

Theoretical molecular weights
Ll-AbiK monomer: 71.6 kDa
Ll-AbiK hexamer: 429.3 kDa
Abi-P2 trimer: 191.6 kDa

Supplementary Figure S9. **Gel filtration/multi-angle light scattering (GF/MALS) analysis of *Ll*-AbiK variants and Abi-P2**. Elution traces from a Superdex 200 Increase 10/300 GL column are shown: absorbance at 280 nm (UV280, blue) and light scattering (LS, red). The values of molecular weight estimates (MW) for the dominant peaks (indicated with arrows and numbers) are given below the graphs.

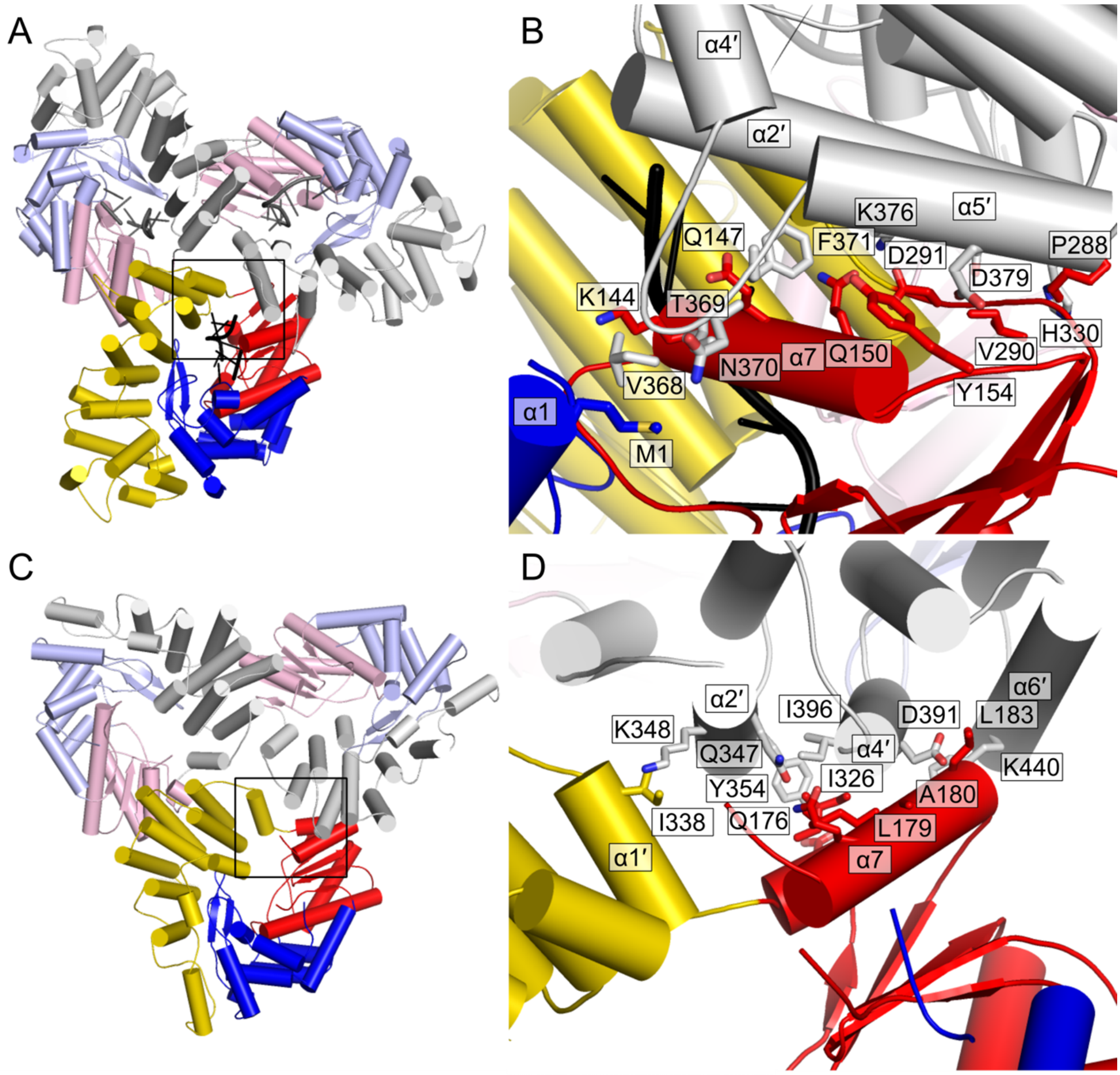

Supplementary Figure S10. **Interaction between protomers within *Ll*-AbiK and Abi-P2 trimers.** (**A**) Interface between *Ll*-AbiK protomers. Protein subdomains are color-coded (fingers, blue and light blue; palm, red and pink; helical domain, yellow and gray). (**B**) Close-up of the boxed region in (A) (different orientation). Residues forming the interface are shown as sticks and colored according to the protomer to which they belong. (**C**, **D**) Interface between Abi-P2 protomers. Color-coding as in (A).

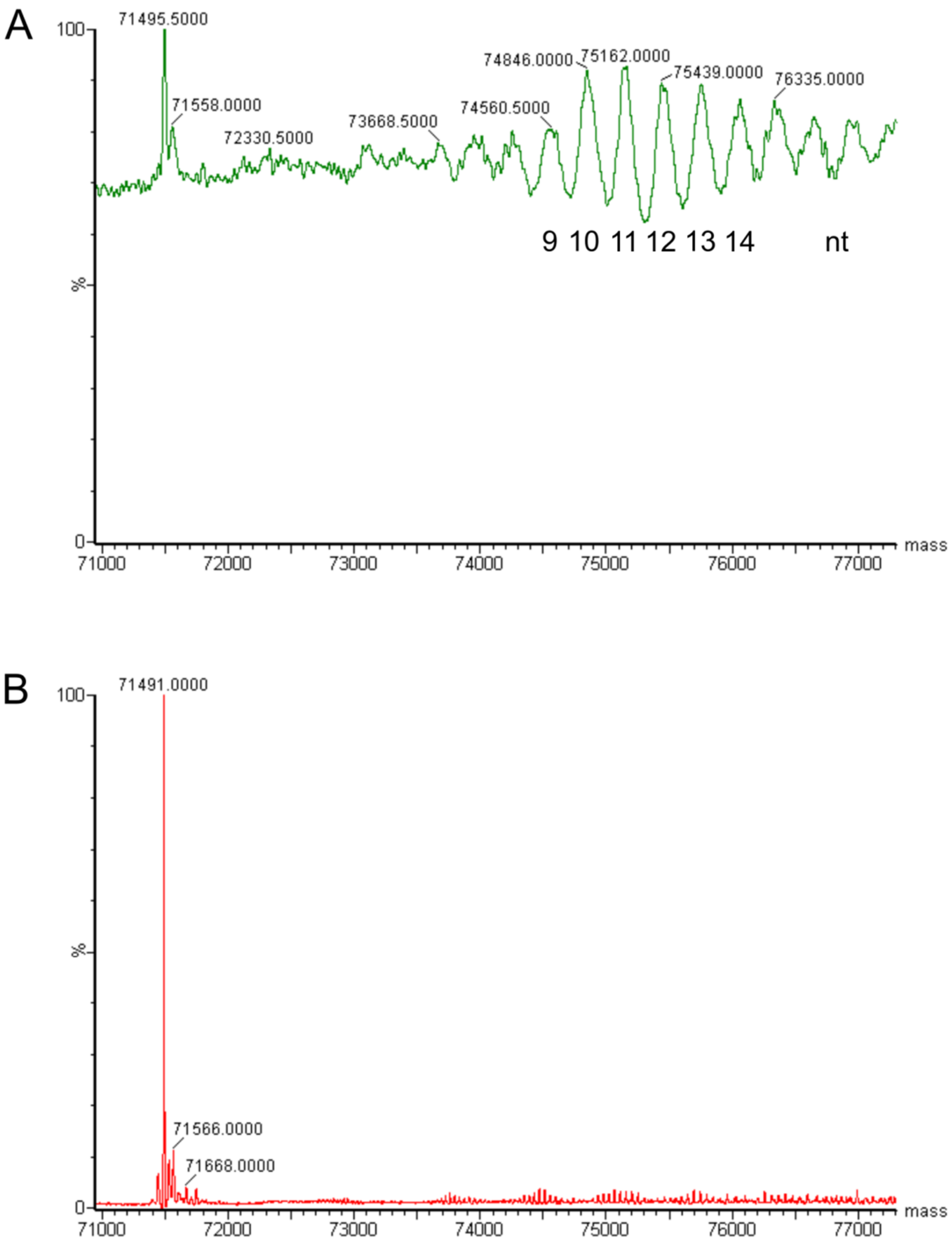

Supplementary Figure S11. **Mass-spectrometry analysis of *Ll*-AbiK variants.** Wild-type *Ll*-AbiK (**A**) and catalytically inactive variant D247N (**B**) were analyzed by LC-MS. Measured molecular weights (Da) are given on top of the peaks. Calculated length of the DNA covalently attached to the enzyme is indicated below the major peaks.

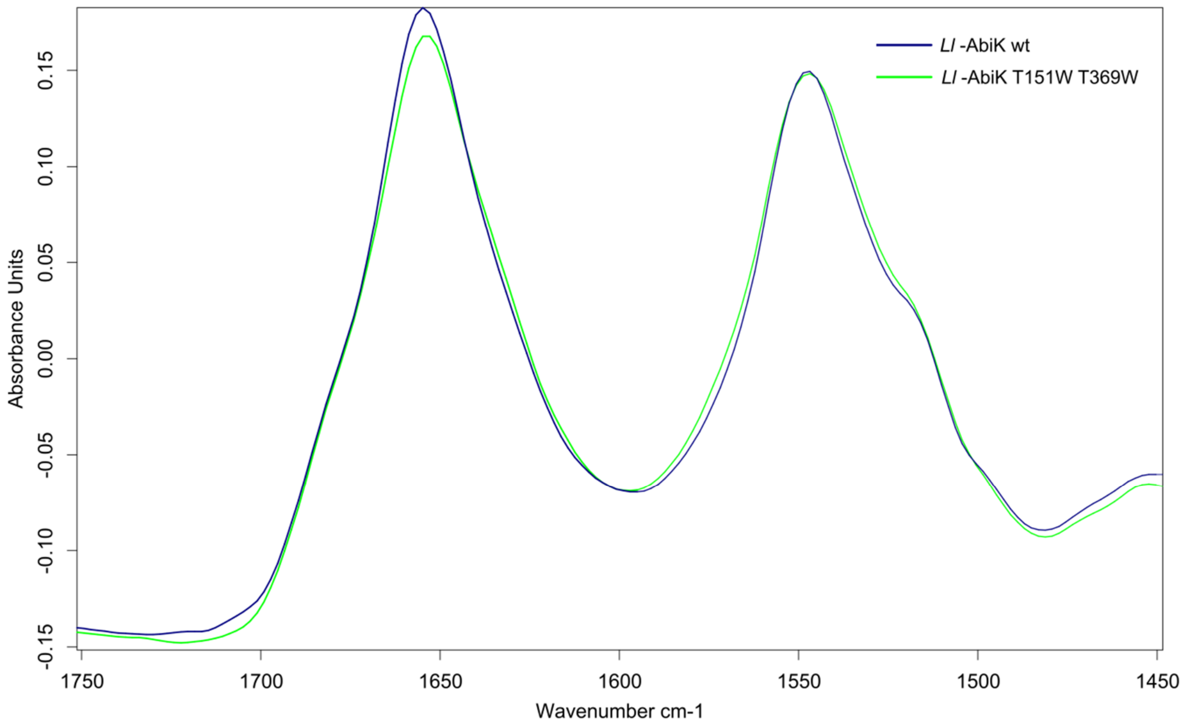

Supplementary Figure S12. **Secondary structure content of wild-type *Ll*-AbiK and T151W/T369W variant.** FT-IR spectra of wild-type *Ll*-AbiK and T151W/T369W variant are shown in blue and green, respectively.

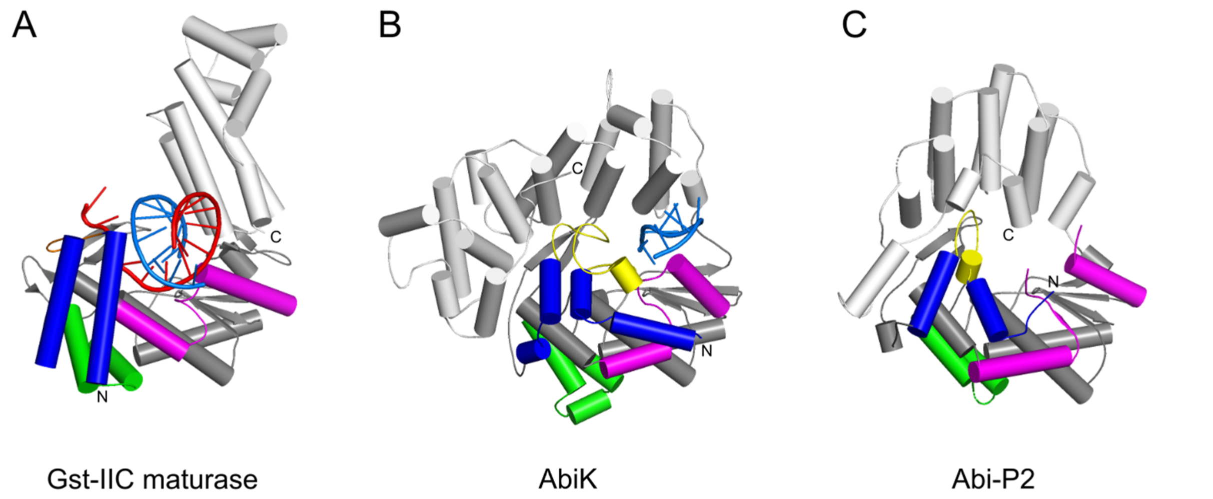

Supplementary Figure S13. **Structural motifs shared between maturase, *Ll*-AbiK and Abi-P2.** Structures of *G. stearothermophilus* maturase (**A**), *Ll*-AbiK (**B**), and Abi-P2 (**C**) are shown. Polymerase domains are shown in gray, additional domains are shown in white. Conserved structural motifs within the polymerase domains are color-coded: NTE, blue; RT0, orange; RT2a, magenta; RT3a, green; region replacing RT0 in Abi polymerases, yellow. DNA and RNA strands are shown as cartoon and colored blue and red, respectively.

BIBLIOGRAPHY

42. Pettersen,E.F., Goddard,T.D., Huang,C.C., Meng,E.C., Couch,G.S., Croll,T.I., Morris,J.H. and Ferrin,T.E. (2004) UCSF ChimeraX: Structure visualization for researchers, educators, and developers. *Protein Sci.*, **30**, 70–82.
